# Potential biomarkers and targets of mitochondrial dynamics

**DOI:** 10.1002/ctm2.529

**Published:** 2021-08-09

**Authors:** Liyang Li, Ruixue Qi, Linlin Zhang, Yuexin Yu, Jiayun Hou, Yutong Gu, Dongli Song, Xiangdong Wang

**Affiliations:** ^1^ Zhongshan Hospital, Department of Pulmonary and Critical Care Medicine, Shanghai Institute of Clinical Bioinformatics Shanghai Engineering Research for AI Technology for Cardiopulmonary Diseases Shanghai China; ^2^ Jinshan Hospital Centre for Tumor Diagnosis and Therapy Fudan University Shanghai Medical College Shanghai China

**Keywords:** lung diseases, mitochondria, mitochondrial dynamics, mtDNA

## Abstract

Mitochondrial dysfunction contributes to the imbalance of cellular homeostasis and the development of diseases, which is regulated by mitochondria‐associated factors. The present review aims to explore the process of the mitochondrial quality control system as a new source of the potential diagnostic biomarkers and/or therapeutic targets for diseases, including mitophagy, mitochondrial dynamics, interactions between mitochondria and other organelles (lipid droplets, endoplasmic reticulum, endosomes, and lysosomes), as well as the regulation and posttranscriptional modifications of mitochondrial DNA/RNA (mtDNA/mtRNA). The direct and indirect influencing factors were especially illustrated in understanding the interactions among regulators of mitochondrial dynamics. In addition, mtDNA/mtRNAs and proteomic profiles of mitochondria in various lung diseases were also discussed as an example. Thus, alternations of mitochondria‐associated regulators can be a new category of biomarkers and targets for disease diagnosis and therapy.

## Graphical abstract

1. The mitochondria quality control system includes mitochondrial dynamics and mitophagy, which is closely related to cellular mesenchymal activations and metabolism reprogramming.

2. Mitochondria can contact with other organelles by the predominant interface of the outer membrane of mitochondria with endoplasmic reticulum, lysosomes, and lipid droplets.

3. The regulation of mitochondrial DNA/RNA transcription and post‐transcriptional modifications are closely related to physiological and pathological processes in various diseases.

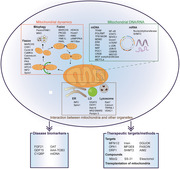

## 1 INTRODUCTION

Mitochondria play crucial roles in cellular metabolism and signaling pathways related to cell death, senescence, and immunity.^1^ The mitochondrial dysfunction widely covers altered ATP production, proton gradient, membrane potential, aberrant complex formation, electron transfer, biogenesis, as well as mitochondrial fusion and fission. Mitochondrial dysfunction contributes to the imbalance of cellular homeostasis and the development of diseases by altering mitochondrial quality controls, interaction with organelles, and expression of mitochondrial DNA/RNA (mtDNA/mtRNA). With the rapid development of methodologies on mitochondrial sequencing, mass spectrum, and omics, the information of disease‐related mitochondrial profiles of genes, proteins, pathways, and regulations can be archived from the further data mining.

MitoCarta (http://www.broadinstitute.org/mitocarta) consists of 1136 human genes and 1140 mouse genes encoding proteins with the information of mitochondrial localizations, submitochondrial compartments, and pathway annotations.^2^ The mitochondrial metabolism‐related gene sets were used to select the nicotinamide nucleotide transhydrogenase as a mediator between hypoxia‐inducible factor 2α and tumor cells “slimming.” Hypoxia‐inducible factor 2α enhanced the expression of miR‐455‐5p and suppressed mediator expression by binding to the 3′ untranslated region.^3^ The MitoMiner mainly contains the information on mammalian mitochondrial localizations, phenotypes, and diseases. The Integrated Mitochondrial Protein Index (IMPI) includes 1330 proteins located on mitochondria, 328 proteins affecting mitochondrial function, morphology and dynamics, and 511 proteins with strong evidence for mitochondrial localizations. The MitoProteome is an object‐relational mitochondrial gene/protein sequence database and annotation system, including 1705 genes and 3625 proteins. Mitochondria‐specific human genes from MitoCarta 3.0 and IMPI database are summarized in Supplemental Table 1, as a candidate list of potential therapeutic targets and diagnostic biomarkers.

The aim of the current review is to explore potential biomarkers and therapeutic targets originated from regulators and molecular mechanisms in mitochondrial processes, with high focuses on the mitochondrial quality control system, interaction between mitochondria and other organelles, and regulation of mtDNA/mtRNA.

## 2 MITOCHONDRIAL QUALITY CONTROL SYSTEM

**FIGURE 1:**
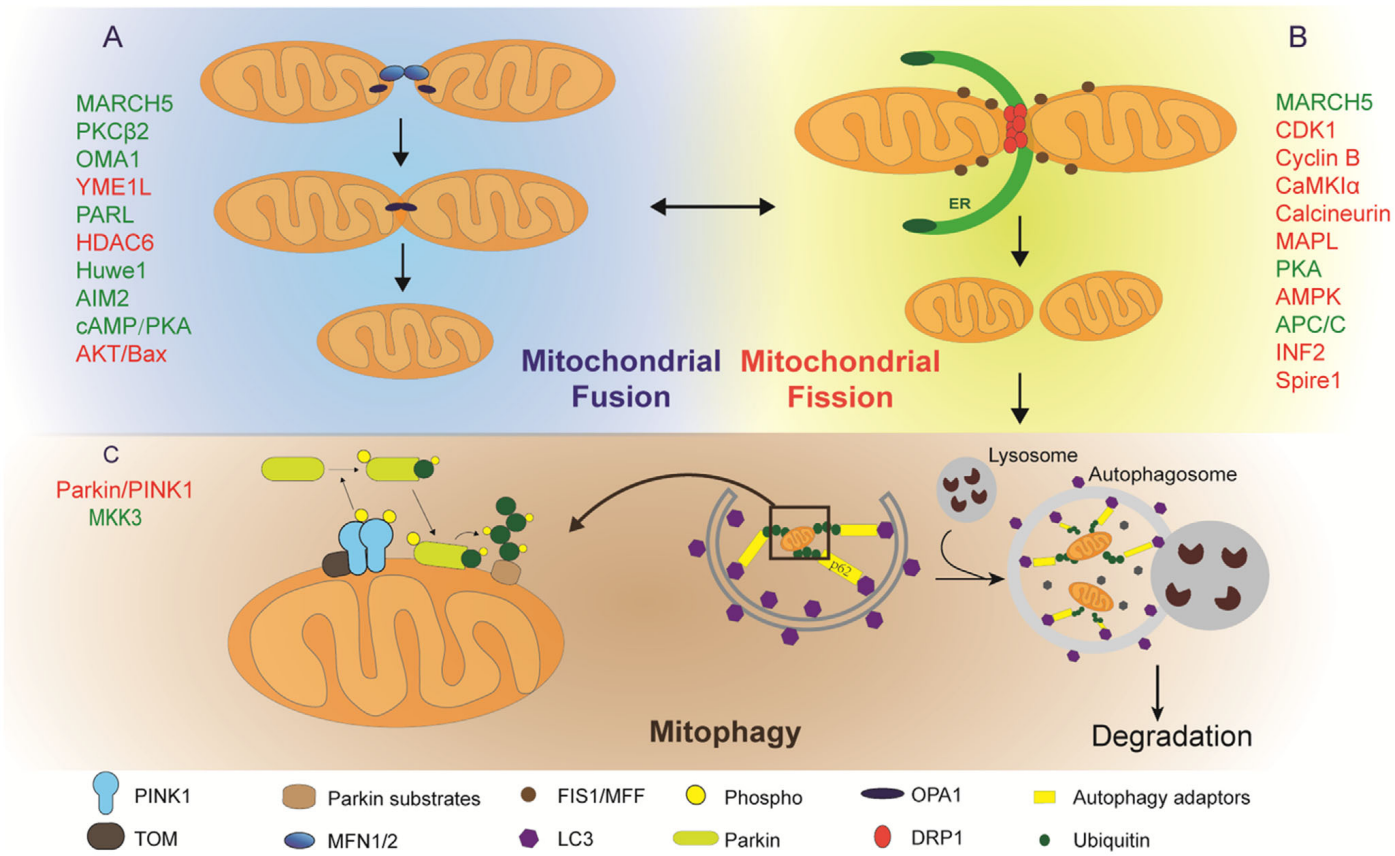
Mitochondria quality control system includes mitochondrial fusion, fission, and mitophagy. A, The process of mitochondrial fusion. B, The process of mitochondrial fission. C, The process of mitophagy, during which the Parkin/ PINK1 pathway plays a crucial role. Related regulatory factors during mitochondrial fusion, fission, or mitophagy were marked in red (positively regulate) or green (negatively regulate) color

The mitochondria quality control system includes mitochondrial dynamics and mitophagy, regulated by several genes, transcriptional factors, proteins, and reactive oxygen species (ROS). As one of highly dynamic organelles, mitochondrion changes the morphology constantly by the dynamic process of fusion and fission (Figure 1) to adapt to various cellular and tissue demands.^4^ Mitochondria interact through oligomerized mitofusin 1/2 protein (MFN1/2), which is located on the outer membrane of mitochondria, and merge into one through optic atrophy type 1 (OPA1) on the inner membrane. MFN1 and MFN2, transmembrane GTPases, share 63% homology with the same functional domains.^5^ MFN1 has a greater activity of GTP‐dependent membrane tethering, while MFN2 with an additional N‐terminal Ras‐binding domain plays more crucial roles in mitochondria‐endoplasmic reticulum (ER) contact site tethering.^6^, ^7^ Mitochondrial fusion is modulated by Smad2, cyclic adenosine 3′,5′‐monophosphate/protein kinase A signaling pathway, and serine/threonine kinase (protein kinase B)/B‐cell lymphoma‐2 associated X phosphorylation (Figure 1A).^8^ During the process of mitochondrial fission, dynamin‐related protein 1 (DRP1) is recruited at actin‐mediated and ER‐mediated mitochondrial construction sites and interacts with mitochondrial fission factors like mitochondrial fission protein 1 (Figure 1B).

**FIGURE 2:**
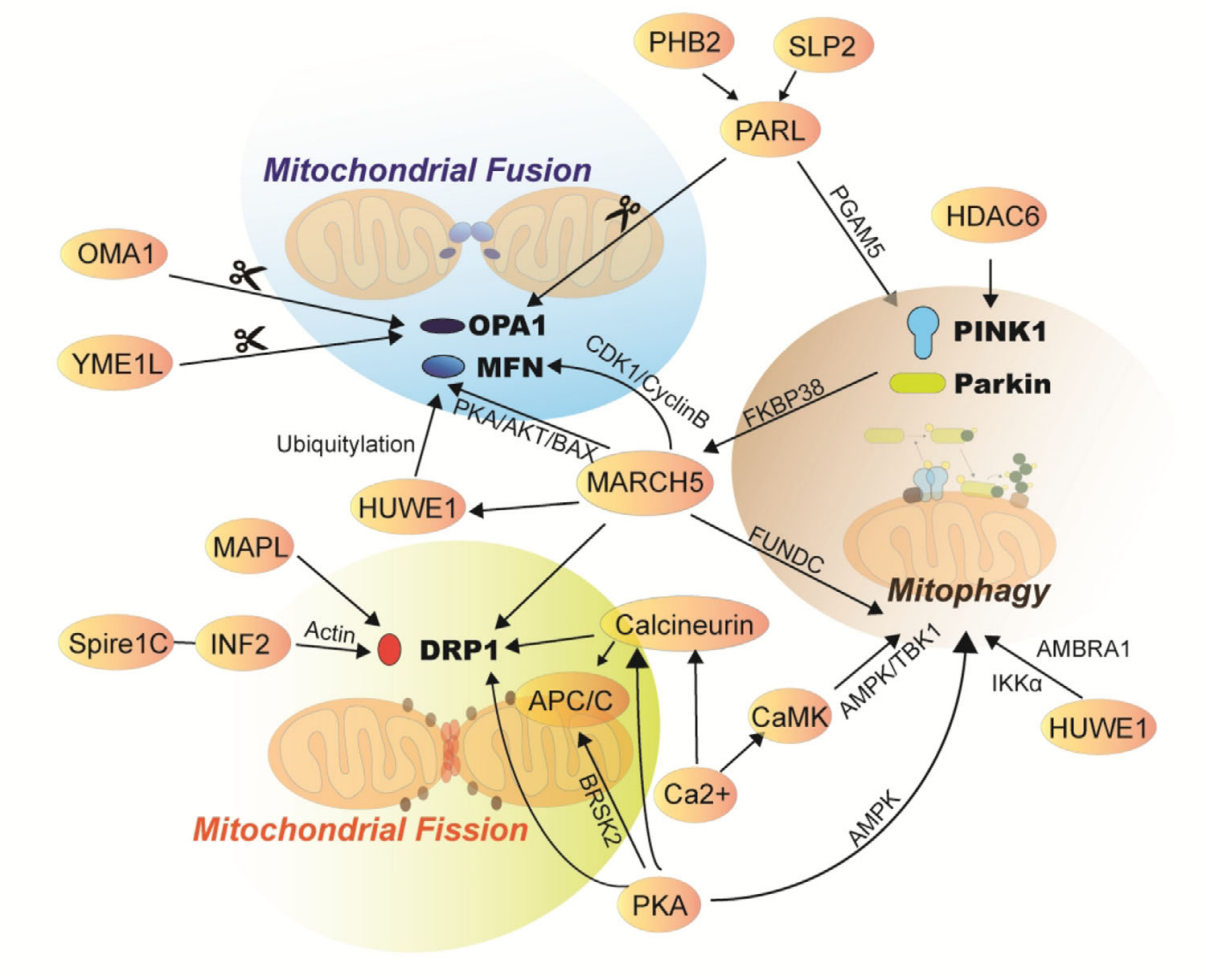
The mitochondrial control system can be regulated by multiple dimensional molecular networks. OMA1, YME1L, and PARL are inner membrane proteases to regulate mitochondrial fusion by cleaving the OPA1. HUWE1, MARCH5, and Parkin are ubiquitin‐ligase enzymes to contribute to mitophagy or directly affected the activity of MFN1/2 or DRP1. In addition, DRP1 could be regulated by INF2, and spire1C, MAPL, calcineurin, which was related to mitochondrial fission and mitophagy. PKA/AMPK signaling pathway could regulate PINK/Parkin and affect mitophagy

Several proteins could interact to regulate mitochondrial dynamics (Figure 2). Of those, the membrane‐associated ring‐CH‐type finger 5 is involved in mitochondrial fusion and fission, while other proteins listed in Figure 1 mainly contribute to the process of either fusion or fission, respectively. The inner mitochondrial membrane proteases regulate mitochondrial fusion by cleaving the OPA1, including OMA1 zinc metallopeptidase, YME1‐like 1 ATPase, and presenilin associated rhomboid like protein.^9^ The ubiquitin‐ligase enzymes contribute to mitophagy or directly affect the activity of MFN1/2 or DRP1, including HECT, UBA, and WWE domain containing 1, membrane‐associated ring‐CH‐type finger 5, and Parkin.^10^, ^11^ NAD^+^‐dependent deacetylases of the sirtuin family (e.g., SIRT3, SIRT4, and SIRT5) are located at mitochondria and regulate mitochondrial dynamics and metabolism in cardiometabolic diseases, Alzheimer's disease, and diabetes.^12^, ^13^, ^14^, ^15^ SIRT3 deficiency was severely disable to the release of mitochondrial cytochrome C to the cytoplasm and led to the remodeling of OPA1‐mediated mitochondrial dynamics.^16^ SIRT5 regulated ammonia‐induced autophagy and mitophagy by controlling glutamine metabolism.^17^ Signaling pathways of extracellular regulated protein kinases, protein kinase A, and c‐Jun N‐terminal kinase were reported to regulate mitochondrial dynamics.^18^, ^19^, ^20^ Signal transducer and activator of transcription 3 (STAT3) acts as an important cytoplasmic transcription factor and regulates activities of glycolytic and oxidative phosphorylation (OXPHOS) by a Ras‐dependent oncogenic transformation.^21^ Phosphorylation of STAT3 in mitochondria could increase mitochondrial membrane potential and OXPHOS activity.^22^, ^23^ STAT3 could be bound to the promotor region of OPA1 and increase its transcriptional level, leading to the changes of mitochondrial morphology and function.^24^ The imbalance of mitochondrial dynamics caused the accumulation of damaged mitochondria, leading to aging, cardiac dysfunction, or cancer. Regulation of mitochondrial fission is able to restore the youthful phenotype of endothelial progenitor, induce the exhaustion of cancer stem cells, and prevent from tumor reoccurrence.^25^, ^26^

Mitophagy as part of the mitochondrial quality control system is one of selective autophagies where damaged mitochondria are degraded, leading to multiple degenerative diseases.^27^ Phosphatase and tensin homolog, which are deleted on chromosome ten induced putative kinase 1 (PINK1)/Parkin pathway, plays an essential role in the development of mitophagy, as detailed in Figure 1C.^28^ PINK1 is degraded by proteases, dependent on the membrane potential of mitochondria. PINK1 is accumulated on the outer membrane of mitochondria where PINK1 interacts with translocases in compromised mitochondria.^29^ Dimerization and cross‐phosphorylation among PINK1 proteins can phosphorylate Parkin and convert mitochondrial ubiquitin chains into Parkin receptors on the mitochondria. The positive feedback loop constructs with PINK1 and Parkin coats can damage mitochondria by binding phospho‐ubiquitin chains with mitophagy adaptors.^29^ Those adaptors recruit autophagosomes, via binding microtubule‐associated protein 1A/1B‐light chain 3, to promote mitophagy. Targeting mitophagy was reported to be a potential therapeutic strategy in chronic obstructive pulmonary diseases (COPD), idiopathic pulmonary fibrosis (IPF), lung injury, and lung cancer.^30^, ^31^, ^32^, ^33^, ^34^ In the pathogenesis of COPD, cigarette smoke could induce mitophagy in lung epithelial cells, leading to cell necroptosis and lung emphysema.^34^ In IPF, disrupted mitophagy could lead to mitochondrial depolarization and promote lung fibrosis.^35^

The mitochondrial dynamics are closely associated with mesenchymal activations, including stem cell maintenance, epithelial‐mesenchymal transition (EMT), or mesenchymal metabolism, to determine stem cell proliferation, migration, differentiation, apoptosis, or aging.^41^ Decreased mitophagy by inhibiting mitochondrial fission could result in the replicative senescence of mesenchymal stem cells.^42^ The EMT of cancer cells by aerobic glycolysis reprogramming could induce mitochondrial fusion through the peroxisome proliferator‐activated receptor γ coactivator‐1α‐ MFN1 pathway, and it could be inhibited by the induction of mitochondrial fusion by overexpression of MFN1.^43^, ^44^ The overexpression of DRP1 increased the development of EMT and migration and invasion of endometrial cancer by altering glucose metabolism.^45^ In liver fibrosis, oxidative stress‐induced mitochondrial fission dysfunction led to hepatocyte EMT by the downregulation of peroxisome proliferator‐activated receptor γ coactivator‐1α.^46^ The mitochondrial dynamics controls the immune cell differentiation by regulating metabolic programming, including aerobic glycolysis or catabolic pathways like fatty acid oxidation.^47^, ^48^ Mitochondrial morphology varied with metabolic needs and was associated with amino acid and lipid metabolism.^49^ The nonessential amino acid serine deprivation could lead to the formation of mitochondrial fragmentations through ceramide metabolism. MFN1/2 regulated the synthesis of phospholipids and cholesterol of lung alveolar epithelial cells in IPF.^50^ The inhibition of DRP1 by particularly interesting new cysteine‐histicline rich protein‐1 resulted in mitochondrial fragmentation and proline synthesis and promoted cell proliferation in lung adenocarcinoma.^51^ Thus, it can be an alternative way to identify and develop diagnostic biomarkers and therapeutic targets for diseases in the process of mitochondrial dynamics‐associated cellular metabolic reprogramming.

## 3 INTERACTION BETWEEN MITOCHONDRIA AND OTHER ORGANELLES

Mitochondria can contact with other organelles by the predominant interface of the outer membrane of mitochondria with ER, lysosomes, endosomes, and lipid droplets (LDs).^52^ The mitochondrial fission requires ER with ER‐bound inverted formin 2 and mitochondrial Spire1C to wrap mitochondria.^53^ Disruption of mitochondria‐ER contact sites affects mitochondrial dynamics, inhibits the occurrence of autophagy, and reduces fatty acid oxidation and OXPHOS.^54^ Peridroplet mitochondria anchored to the LDs reduced of the motility and regulation of lipid metabolism via bidirectional signaling.^55^ Acyl CoA:diacylglycerol acyltransferase 2‐ fatty acid transport protein 1 and Rab18‐NRZ/SNARE complexes are integrated on the membrane responsible for interactions between LDs and mitochondria. The LDs interact with mitochondria through perlipin 5 to maintain the function and stability of regulations among mitochondrial dynamics and lipids, cytoplasmic homeostasis, and cell structure and function. The intercommunication between intracellular organelles is responsible for the production of mitochondria‐driven lipids, which enable to conversely maintain the interaction between mitochondria and other organelles. LDs are essential for the regulatory process of mitochondria dynamics‐associated lipid metabolism when mitochondria and organelles signal each other.^56^ The interaction between mitochondria and lysosome modulates the process of mitochondrial dynamics and facilitates the transfer of metabolites or ions.^57^, ^58^ Mitochondria‐lysosome contact sites tethering is regulated by mitochondrial Rab7 GTP hydrolysis and regulates mitochondrial calcium dynamics and transport calcium to mitochondria through the lysosomal calcium efflux channel and transient receptor potential mucolipin 1.^57^ Mitochondrial calcium uptake at mitochondria‐lysosome contacts is modulated by outer and inner mitochondrial membrane channels, voltage‐dependent anion channel 1, and mitochondrial calcium uniporter, respectively. Interactions among ER, endosomes, and mitochondria regulate lipid transport and endosomal function and maturation.^59^ Mitochondria are recruited at contact sites where the ER transmembrane protein, PDZ domain containing 8, interacts with Rab7‐GTP and another ER protein protrudin.^59^ The lipid transfer domain of the PDZ domain containing eight functions as a conduit for phospholipid transfer among the ER, endosomes, and mitochondria, to drive endosomal maturation. The interaction between mitochondria and subcellular compartments is essential in various aspects of physiological and pathological activities, including metabolism, immunity, and cell death.^60^

## 4 MITOCHONDRIAL DNA

**FIGURE 3:**
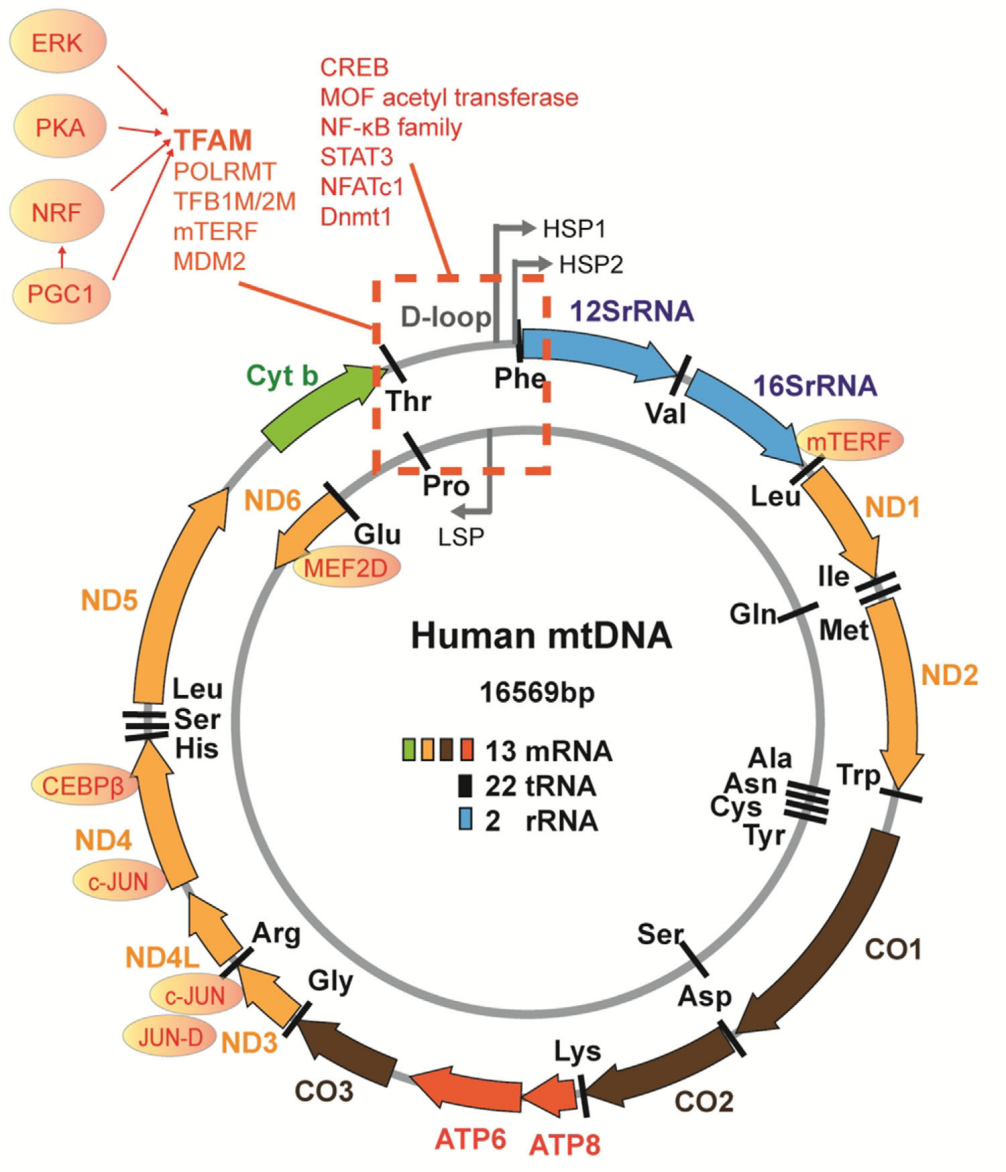
Human mtDNA is consisted of 16569 bp, including heavy (H) strand and light (L) strand. H strand (the outer circle in gray) encodes 12mRNAs, 14tRNAs, and two rRNAs, and L strand (the inner circle in gray) encodes one mRNA and eight tRNAs. The H strand promoter HSP1, HSP2, and L strand promoter LSP are located in the D‐loop region. mtDNA does not have introns and the coding region of each gene is closely connected to each other or even partially overlapped. Regulatory elements of mtDNA transcription were marked in red color. The direct regulators include the mitochondrial RNA polymerase (POLRMT), two transcription factors ‐mitochondrial transcription factor A (TFAM) and mitochondrial transcription factor B1/2 (TFB1M/2 M), one transcription elongation (TEFM), and one known transcription termination factor (mTERF1). cAMP response element‐binding protein (CREB) binds to D‐loop to promotes mtDNA gene expression. Myocyte enhancer factor 2D (MEF2D), c‐Jun, JunD, and CCAAT enhancer‐binding protein (CEBP)β can also bind to the different regions in mtDNA. Besides, extracellular regulated protein kinases (ERK1/2), protein kinase A (PKA), peroxisome proliferator‐activated receptor γ coactivator (PGC1) family, and nuclear factor E2‐related factor (NRF)1/2 could indirectly regulate the mtDNA transcription, targeting TFAM, which is a crucial factor in mtDNA transcription

**FIGURE 4:**
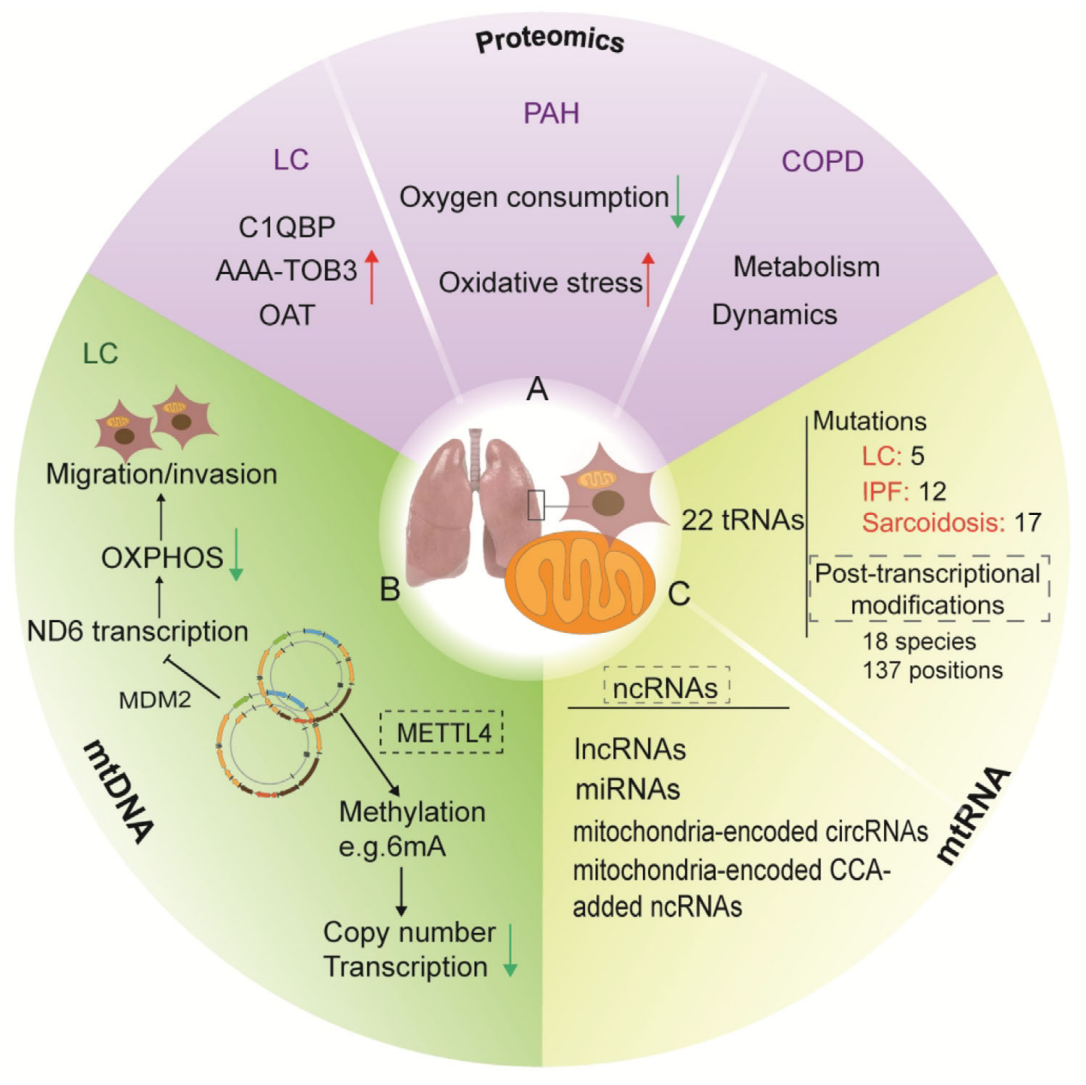
Conclusion of proteinomics, mtDNA, and mt‐RNA in lung diseases. A, Proteinomics of lung cancer (LC), pulmonary artery hypertension (PAH), and chronic obstructive pulmonary diseases (COPD) patients or cell models by liquid chromatography–tandem mass spectrometry (LC‐MS/MS) found many specific proteins related to mitochondrial function. In lung cancer, complement C1q‐binding protein (C1QBP), ornithine aminotransferase (OAT), and ATPase family AAA domain‐containing protein 3B (AAA‐TOB3) were upregulated and correlated to clinical outcomes. In PAH or COPD, proteins of different expression with the healthy could be clustered into mitochondrial pathway including oxygen consumption, oxidative stress, metabolic reprograming, and mitochondrial dynamics. B, The studies on mtDNA in LC found that mouse double minute 2 proto‐oncogene could promote cancer cell migration and invasion through its regulation of mtDNA transcription. Posttranscriptional modification of mtDNA such as 6 mA methylation could repress DNA binding and bending by methyltransferase like 4 (METTL4), which is a lack of studies in lung diseases. C, A few studies on mt‐tRNA in LC, idiopathic pulmonary fibrosis (IPF), and sarcoidosis found 5,12, and 17 tRNA mutations, respectively. Noncoding RNAs in mitochondria are also important, but no studies were conducted in lung diseases. The dotted box represented the lack of studies in lung diseases

Human mitochondria contain 16569 bp mtDNA, heavy strands encoding 12 mRNAs, 14 transfer RNAs (tRNAs), 2 ribosomal RNA (rRNAs), and light strands encoding one mRNA and eight tRNAs, as detailed in Figure 3. The close connection and partial overlap are biological characters of mtDNA gene coding regions. mtDNA‐encoded 13 proteins are core constituents of mitochondrial electron transport chain complexes. Twenty‐two tRNAs and two mitochondrial rRNAs are essential components for the mitochondrial translational apparatus, as explained in Figure 3 and listed in Table 1.^4^ Many proteins either directly bind or indirectly regulate the mtDNA transcription (Figure 3), of which the mitochondrial transcription factor A (TFAM) stabilizes mtDNA and initiates the transcription. In human lung cancer, mouse double minute 2 proto‐oncogene could inhibit the transcription of mitochondrial ND6 by binding mtDNA, as a specific subunit of the electron transport chain, leading to defects in the electron transport chain (Figure 4B).^61^ Among regulatory elements of mtDNA, extracellular regulated protein kinases 1/2, protein kinase A, peroxisome proliferator‐activated receptor γ coactivator family, and nuclear factor E2‐related factor 1/2 indirectly regulated the mtDNA transcription by targeting TFAM. cAMP response element‐binding protein promotes mtDNA gene expression by binding to cyclic AMP response elements in the D‐loop region. Decreased activity of cAMP response element‐binding protein results in mitochondrial respiration dysfunction by downregulating expression of many mitochondrial genes like ND5.^62^ The myocyte enhancer factor 2D regulated the mtDNA transcription through binding to MEF2 consensus site encoding ND6.^63^ c‐Jun, JunD, and CCAAT enhancer‐binding protein βbind to different regions in mtDNA, as showed in Figure 3.

**TABLE 1:** 13 mRNAs encoded by mtDNA

Gene name	Start	End	The function of encoding protein
ATP6	8527	9207	Subunits of respiratory Complex V
ATP8	8366	8572	Subunits of respiratory Complex V
ND1	3307	4262	Subunits of respiratory Complex I
ND2	4470	5511	Subunits of respiratory Complex I
ND3	10059	10404	Subunits of respiratory Complex I
ND4	10760	12137	Subunits of respiratory Complex I
ND4L	10470	10766	Subunits of respiratory Complex I
ND5	12337	14148	Subunits of respiratory Complex I
ND6	14673	14149	Subunits of respiratory Complex I
CO1	5904	7445	Constituents of Complex IV
CO2	7586	8269	Constituents of Complex IV
CO3	9207	9990	Constituents of Complex IV
CytB	14747	15887	Components of Complex III

**FIGURE 5:**
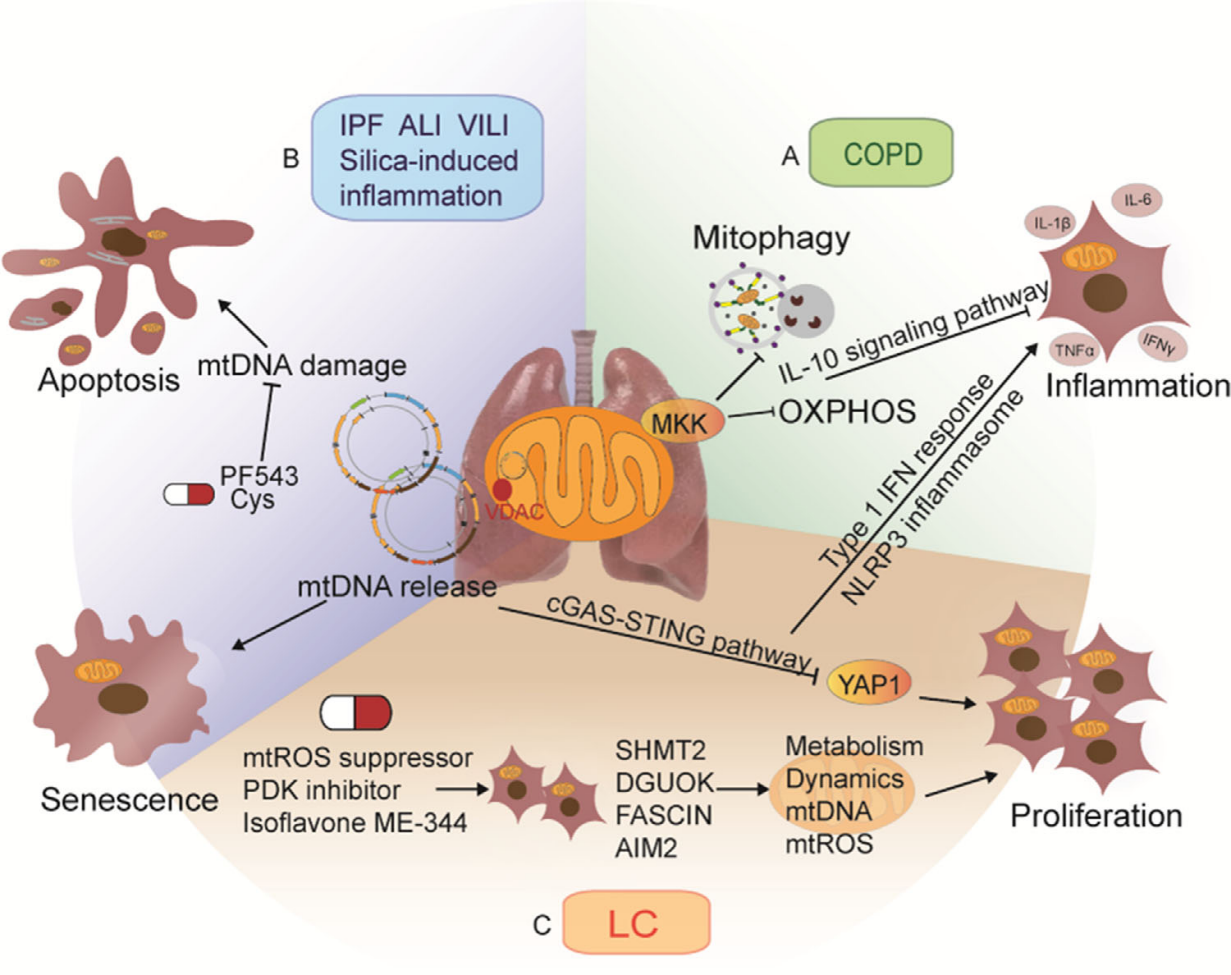
Different lung diseases including chronic obstructive pulmonary diseases (COPD), idiopathic pulmonary fibrosis (IPF), acute lung injury (ALI), ventilator‐induced lung injury (VILI), silica‐induced lung inflammation, and lung cancer (LC) have effects on mitochondria, leading to changes of cell biological function such as cell death, inflammation, senescence, proliferation. A, In COPD, deletion of mitogen‐activated protein kinase 3 (MKK3) could promote mitophagy and oxidative phosphorylation to activate the IL‐10 signaling pathway and inhibit inflammation. B, In other inflammatory pulmonary diseases, mtDNA release was identified to activate downstream signaling pathway including cyclic GMP–AMP synthase (cGAS), stimulator of interferon genes (STING), NOD‐like receptors (NLRP3) inflammasome, and Yes‐associated protein 1 (YAP1) and exert effects on cell senescence, proliferation, and inflammation. C, In lung cancer, genes including mitochondrial deoxyguanosine kinase (DGUOK), Fascin, serine hydroxymethyltransferase 2 (SHMT2), absent in melanoma 2 (AIM2) and potential medications including mtROS inhibitor, phosphoinositide dependent protein kinase (PDK) inhibitor, isoflavone ME‐344 could affect mitochondrial function such as metabolism, mtDNA stability, and integration, ROS generation and mitochondrial dynamics

Environmental factors or cell endogenous stresses induce the fragmentation and damage of mtDNA, activating inflammatory signaling pathways and consequently leading to immune cell dysfunction and death.^64^ The release of mtDNA and activation of downstream signaling pathways are controlled by various genes and chemical compounds, including cyclic GMP–AMP synthase, stimulator of interferon genes, and Yes‐associated protein 1. In pathological conditions, mtDNA was passed through formed pores in mitochondrial outer membranes on basis of voltage‐dependent anion channels oligomers and released to cytosol to activate the pathway of cyclic GMP–AMP synthase stimulator of interferon genes and influence cell proliferation, senescence, and inflammation (Figure 5B).^65^, ^66^, ^67^, ^68^, ^69^ In antimicrobial innate immunity, mtDNA as a proinflammatory or inflammasome agonist influenced immune responses and inflammatory pathology.^70^ MtDNA released into extracellular spaces can generate neutrophil extracellular traps and elevate type I interferon response.^71^ Sphingosine kinase 1 inhibitor or cyclosporine‐A reduced lung injury and fibrosis by alleviating mtDNA damage.^72^, ^73^ Higher concentrations of circulating mtDNA were correlated with poor prognosis of patients with IPF, sepsis, or ICU treatment.^74^, ^75^, ^76^ Patients with more extracellular mtDNA in sarcoidosis had the higher frequency of extrapulmonary diseases.^77^ These studies suggest that mtDNA can be a useful biomarker in diseases.

The formation and accumulation of aberrant mtDNA variants, including mutations, deletions, and linear molecules, were found to contribute to the occurrence of mitochondrial diseases, such as MELAS syndrome or Kearns Sayre syndrome.^78^ CRISPR‐free DddA‐derived cytosine base editing was applied for the precise manipulation of mtDNA with high target specificity and product purity.^79^ Gene modification and editing on mtDNA were suggested to be potential therapeutic approaches for mitochondrial diseases, mitochondria‐associated tissue damage, and organ failure, though there are still challenges in the delivery of guide RNA into mitochondria during mtDNA base editing by CRISPR‐Cas9 system.^80^, ^81^, ^82^, ^83^ Mammalian mtDNA was enriched for *N*6‐methyldeoxyadenosine (6 mA), which contributed to attenuated mtDNA transcription and a reduced mtDNA copy number.^84^ The methylation of the mitochondrial genome was also demonstrated in other studies, challenging the notion that mtDNA was lowly methylated.^85^, ^86^ Posttranscriptional modifications of mtDNA were identified in mtDNA CpG island and light‐strand non‐CpG.^87^, ^88^ 6 mA site density and expression in mtDNA were about more than 8000 times higher than in the nuclear genome.^89^ DNA methylation regulates human mitochondrial stress response through the activation of methyltransferases. mtDNA 6 mA methylation repressed DNA binding and bending by methyltransferase like 4 and TFAM (Figure 4B).^84^ Patterns and strand biases of mtDNA regional methylation were dynamically regulated by DNA methyltransferase 3A. The degree of mtDNA methylation differs in species, developing stages, ages, and diseases. Methyltransferases, nucleotides, mt‐RNAs, and other epigenetic modifications also regulate the methylation‐associated network and interaction with genome DNA.^88^

## 5 MITOCHONDRIAL RNA

Posttranscriptional modifications of mitochondrial tRNA (mt‐tRNA) are important in the maintenance of mitochondrial function, including 18 species of modified nucleosides at 137 positions in 22 mt‐tRNAs. mt‐RNAs are added with CCA nucleotides by tRNA nucleotidyltransferase, a CCA‐adding enzyme, as shown in Figure 4C.^90^, ^91^ m1A and m1G were related to clinical prognosis in 12 cancer types, including lung adenocarcinoma and lung squamous cell carcinoma.^92^ Serine hydroxymethyl transferase 2 provided methyl donors and produced the taurinomethyluridine base at the wobble position of selected mitochondrial tRNAs, responsible for mtDNA translation and mitochondrial respiratory chain function.^93^, ^94^ The sequencing of mt‐tRNAs demonstrated five specific mutations in lung cancer, including tRNA^Ala^ T5655C, tRNA^Arg^ T10454C, tRNA^Leu^ (CUN) A12330G, tRNA^Ser^(UCN)T7505C, or tRNA^Thr^ G15927A, and 12 or 17 mutations of 22 mt‐tRNA genes in patients with IPF or sarcoidosis (Figure 4C).^95^

Noncoding RNAs (ncRNAs), including microRNAs (miRNAs), long noncoding RNAs (lncRNAs), and circular RNAs (circRNAs), are involved in the regulation of mitochondrial oxidative metabolism and energy supply, and also act as messengers between mitochondria and nucleus.^96^, ^97^ Mitochondrial ncRNAs, including nuclear‐genome‐encoded ncRNAs located in mitochondria and mtDNA‐encoded ncRNAs, regulate glycolysis, energy status, gene expression, metabolism, and mtDNA transcription .^98^, ^99^ Of those, miR‐181c downregulates the expression of cytochrome C oxidase subunit 1, while upregulated subunit 2 and 3, remodeling the complex IV and overactivating OXPHOS.^100^ LncND5, lncND6, and lncCyt b were discovered within the mitochondrial transcriptome as the counterpart antisense transcripts of the mitochondrial ND5, ND6, and cytochrome b mRNAs, to regulate mitochondrial gene expression.^99^ LncRNA growth‐arrest‐specific 5 as a tumor suppressor maintains cellular energy homeostasis by modulating mitochondrial tricarboxylic acid cycle.^101^ LncRNA 1810058I24Rik coding protein, mitochondrial micropeptide‐47, was also localized on mitochondria and activated NLRP3 inflammasome in innate immunity.^102^ Studies on mitochondrial circRNAs profiling of six subcellular compartments, including nucleus, cytoplasm, mitochondria, ribosome, cytosol, and exosome, in hepatocytes demonstrated 118 circRNAs of low abundance in mitochondria.^103^ Mc‐COX2, one of the mitochondrial genome‐derived circRNAs, maintains the ATP production and mitochondrial membrane potential in chronic lymphocytic leukemia cells.^104^ The sequencing and analysis of RNAs isolated from mitochondria demonstrated that circRNAs were encoded in nuclear or mitochondrial genomes in cell lines of human and mouse (HeLa, HEK293T, RPE‐1, HepG2, N2a, and NIH3T3 cells), with 248 and 268 mtDNA‐encoded circRNAs.^105^ Two mecciRNAs, mecciND1, and mecciND5, were proposed to interact with complexes such as TOM40 or purine nucleoside phosphorylase and facilitated the mitochondrial entry of nuclear‐encoded proteins by serving as molecular chaperones in the folding of imported proteins.^105^

## 6 CLINICAL SIGNIFICANCE OF MITOCHONDRIAL FUNCTION

The fast‐development of metabolomics and genomics provides new insights of deeper understanding about mitochondrial function, including plasticity, biosynthesis, as well as versatility of tumor metabolism and bioenergetics associated with tumorigenesis and metastasis.^106^ Genes and proteins involved in the regulation of mitochondrial function have been suggested to be therapeutic targets and diagnostic biomarkers for chronic lung diseases, and depend on the disease‐, severity‐, duration‐, and therapy‐specific alterations.^107^ For example, the MFN2 or DRP1 proteins involved in mitochondrial dynamics were reported as potential therapeutic targets for diabetic cardiomyopathy.^108^, ^109^ Factors involved in the process of mitochondrial dynamics can be the candidates of therapeutic targets for adjuvant cancer chemotherapy and biomarkers for age‐related diseases like Alzheimer's disease.^110^, ^111^, ^112^, ^113^ The maintenance of mitochondrial function is an alternative therapeutic strategy for diseases, an approach of bioenergy‐based regulations to improve cell sensitivities to challenges and drugs, and an effective path to cure tissue injury and alleviate clinical phenomes.

The fibroblast growth factor 21 or growth differentiation factor 15 were verified as diagnostic biomarkers in a clinical cohort of patients with mitochondrial diseases.^114^, ^115^ Irisin was proposed to be a potential therapeutic target for sepsis or myocardial ischemia‐reperfusion injury by inhibiting ferroptosis, reducing the inflammatory response, restoring mitochondrial homeostasis, and maintaining ER‐mitochondria interaction through mitochondrial ubiquitin ligase‐dependent mechanism.^116^, ^117^ Milk fat globule EGFR factor 8 ameliorates the severity of pancreatic tissue injury and the mortality of animals with acute pancreatitis by activating the integrin‐containing focal adhesion kinase‐signal transducer and STAT3 pathway to restore mitochondrial NADH dehydrogenase activities, ATP production, and mitochondrial morphology.^118^

The oxidative phosphorylation, mitochondria quality control system, and mtDNA transcription declined in chronic lung diseases and fibrosis, acute lung injury/acute respiratory distress syndrome, lung inflammation, and lung cancer, as one of examples to explain roles of mitochondrial dynamics in the pathogenesis of diseases.^119^, ^120^, ^121^, ^122^ The mitochondria quality control plays an important role in the maintenance of the cellular metabolic stability and organ function by eliminating damaged mitochondria. The imbalance of mitochondrial dynamics occurred in primary alveolar type II cells of patients with COPD and triggered aging‐associated chronic lung diseases.^37^, ^38^ Impaired fusion and fission measured by low levels of MFN1, OPA1, FIS1, and p‐DRP1 were correlated with disease severity. The mitochondrial control system is regulated by multi‐dimensional molecular networks. Mitogen‐activated protein kinase kinase 3 (MKK3), a dual‐specificity protein kinase, participates in innate immune responses, upstream regulation of p38 mitogen‐activated protein kinase pathway, and inflammatory cell recruitment to lungs during inflammation. MKK3 also acts as a critical mediator of mitochondrial ROS generation and regulates the expression of tricarboxylic acid cycle enzymes during inflammatory injury or sepsis.^39^ In addition, MKK3 is decisive in maintaining the function of bone marrow‐derived macrophages and upregulating mitophagy to clean damaged mitochondria, especially in cigarette smoke‐induced lung inflammation (Figure 5A).^40^ The release of mtDNA activates the signaling pathway of cyclic GMP–AMP synthase, stimulator of interferon genes, NOD‐like receptors inflammasome, and Yes‐associated protein 1, contributing to lung cell senescence, proliferation, and inflammation (Figure 5B). In lung cancer, upregulated expression of mitochondrial deoxyguanosine kinase, fascin, and serine hydroxymethyltransferase 2, and downregulated melanoma 2 were correlated with mitochondrial dysfunction.^119^, ^121^, ^123^, ^124^ Lower mitochondrial oxidative phosphorylation, mtDNA instability, ROS generation, and changes in mitochondrial dynamics, often appears in lung cancer, as detailed in Figure 5C.^72^, ^119^, ^121^, ^123^, ^124^, ^125^, ^126^

Proteomic analysis of human normal lung cells and cancer cells showed changes of mitochondrial proteome profiles as cancer subtype‐specific biomarkers or potential therapeutic targets for lung cancer (Figure 4).^127^, ^128^ Anticancer drugs inhibit the proliferation of lung cancer cells through targeting either mitochondrial bioenergetics, mitochondrial ROS production, or pyruvate dehydrogenase kinase activity (Figure 5C).^125^, ^126^, ^129^ Vitamin D3 downregulated mitochondrial ROS and related participants, for example, cyclooxygenase‐2, superoxide dismutase 2, NOD‐like receptor family pyrin domain containing 3, caspase‐1, SIRT3, and interleukin‐1β.^130^ Changes in mitochondrial proteins and functions were also noticed in pulmonary arterial hypertension (PAH) and COPD.^37^, ^131^, ^132^ Disease‐specific proteins related to mitochondrial function were identified from proteomic profiles of lung cancer, PAH, and COPD clinically and experimentally. The complement C1q‐binding protein, ornithine aminotransferase, and ATPase family AAA domain‐containing protein 3B were upregulated and correlated to the prognosis of patients with lung cancer.^127^, ^128^, ^133^ Expressions of proteins of mitochondria‐associated pathways differ among healthy and patients with PAH or COPD, including oxygen consumption, oxidative stress, metabolic reprograming, and mitochondrial dynamics (Figure 4A). It provides evidence that the mitochondrial homeostasis is important in lung morphology and function, though roles of mtDNA/mtRNA/ncRNAs, posttranscriptional modifications of mtDNA/mtRNA, and related regulating factors remain unclear in the development of diseases.

A number of mitochondrial‐targeted compounds have been developed or underdevelopment to improve mitochondrial dysfunction. For example, MitoQ, a mitochondrial antioxidant consisted of a quinone moiety linked to a triphenylphosphonium moiety by a 10‐carbon alkyl chain, protects against mitochondrial oxidative damage in the heart failure, hypertension, or other diseases.^134^ SS‐31 as a mitochondria‐targeted therapeutic agent binds to cardiolipin on the inner mitochondrial membrane and protects the electron carrying function of cytochrome C.^135^ Elesclomol as a mitochondria‐targeted chemotherapeutic compound has the anticancer efficacy in preclinical and clinical testing.^136^ On the other hand, the mitochondrial signal‐retaining autophagy indicator with acid‐fast fluorescent protein tags (TOLLES: tolerance of lysosomal environment) and Ypet are applied to visualize mitophagy. Since TOLLES and Ypet are effectively degraded by mitophagy, their molecular linkage is quantifiable as a new mitophagy indicator for high‐throughput screening of therapeutic mitophagy inducers to target damaged mitochondria.^36^

Transplantation of mitochondria into targeted tissues and cells has been considered as an alternative strategy to restore mitochondrial function and had promising therapeutic effects in cardiovascular and cervical nervous system diseases.^137^, ^138^, ^139^, ^140^ The transplantation of autologously derived mitochondria improves the severity of experimental ischemia‐reperfusion heart injury and cardiac function by targeting cardiomyocytes, enhancing oxygen consumption and high‐energy phosphate synthesis, and promoting the activities of cytokine mediators and proteomic pathways.^141^ The autologous mitochondrial transplantation could prevent the cardiac and pulmonary function of pediatric patients with myocardial ischemia–reperfusion injury with extracorporeal membrane oxygenation therapy.^142^ The transplantation of nonautologous mitochondria from healthy skeletal muscle cells into normal cardiomyocytes provides a short‐term improvement of basal respiration, ATP production, maximal respiration, and spare respiratory capacity.^139^ The placental mitochondrial fractions as a source for mitochondrial transplantation, showed therapeutic effects in experimental cerebral ischemia‐reperfusion injury and diabetes‐associated cognitive impairment.^138^, ^140^ The mitochondrial transplantation can be an alternative to new therapies for clinical trials. However, several critical issues still need to be further clarified, including underlying mechanism, standardization of mitochondrial separation and preservation methods, and maintenance of long‐term therapeutic efficacy.

## 7 CONCLUSION

The present review was specially focused on molecular mechanisms and processes involved in the mitochondrial quality control system, interaction between mitochondria and other organelles, and the regulation of mtDNA/mtRNAs transcription. The process of mitochondria‐dominated and associated metabolism, immunity, cell death, and several signaling pathways can be a new source to develop diagnostic biomarkers and therapeutic targets for clinical and translational medicine. There is a potential for drug discovery and development by interfering mitochondrial dynamics, the interaction between mitochondria and other organelles such as LD, ER, endosomes, lysosomes, and the regulation of mtDNA/mtRNAs transcription and posttranscriptional modifications. Thus, we call special attention to target mitochondrial regulation and involved factors in the pathogenesis of diseases and uncover a new generation of mitochondria‐targeted clinical therapies.

## CONFLICTS OF INTEREST

All authors declare that they have no conflicts of interest.

## Supporting information

Supporting Information
