# Supplementary material for: Potential biomarkers and targets of mitochondrial dynamics
Source: Clin Transl Med. 2021 Aug 9;11(8):e529. doi: 10.1002/ctm2.529 (PMC8351522; doi:10.1002/ctm2.529)
Supplement: Supplementary file 1 — Supporting Information [file CTM2-11-e529-s001.docx]

Supplementary table1:

| Mitochondria-specific human genes from MitoCarta3.0 and IMPI database (1506 genes) | |
| --- | --- |
| Gene Symbol | Description |
| AADAT | aminoadipate aminotransferase |
| AARS2 | alanyl-tRNA synthetase 2, mitochondrial |
| AASS | aminoadipate-semialdehyde synthase |
| ABAT | 4-aminobutyrate aminotransferase |
| ABCA9 | ATP binding cassette subfamily A member 9 |
| ABCB10 | ATP-binding cassette, sub-family B (MDR/TAP), member 10 |
| ABCB6 | ATP-binding cassette, sub-family B (MDR/TAP), member 6 (Langereis blood group) |
| ABCB7 | ATP-binding cassette, sub-family B (MDR/TAP), member 7 |
| ABCB8 | ATP-binding cassette, sub-family B (MDR/TAP), member 8 |
| ABCD1 | ATP binding cassette subfamily D member 1 |
| ABCD2 | ATP binding cassette subfamily D member 2 |
| ABCD3 | ATP binding cassette subfamily D member 3 |
| ABCE1 | ATP-binding cassette, sub-family E (OABP), member 1 |
| ABCF2 | ATP-binding cassette, sub-family F (GCN20), member 2 |
| ABCG2 | ATP-binding cassette, sub-family G (WHITE), member 2 (Junior blood group) |
| ABHD10 | abhydrolase domain containing 10 |
| ABHD11 | abhydrolase domain containing 11 |
| ACAA1 | acetyl-CoA acyltransferase 1 |
| ACAA2 | acetyl-CoA acyltransferase 2 |
| ACACA | acetyl-CoA carboxylase alpha |
| ACACB | acetyl-CoA carboxylase beta |
| ACAD10 | acyl-CoA dehydrogenase family, member 10 |
| ACAD11 | acyl-CoA dehydrogenase family, member 11 |
| ACAD8 | acyl-CoA dehydrogenase family, member 8 |
| ACAD9 | acyl-CoA dehydrogenase family, member 9 |
| ACADL | acyl-CoA dehydrogenase, long chain |
| ACADM | acyl-CoA dehydrogenase, C-4 to C-12 straight chain |
| ACADS | acyl-CoA dehydrogenase, C-2 to C-3 short chain |
| ACADSB | acyl-CoA dehydrogenase, short/branched chain |
| ACADVL | acyl-CoA dehydrogenase, very long chain |
| ACAT1 | acetyl-CoA acetyltransferase 1 |
| ACBD3 | acyl-CoA binding domain containing 3 |
| ACCS | 1-aminocyclopropane-1-carboxylate synthase homolog (inactive) |
| ACLY | ATP citrate lyase |
| ACO2 | aconitase 2, mitochondrial |
| ACOD1 | aconitate decarboxylase 1 |
| ACOT11 | acyl-CoA thioesterase 11 |
| ACOT13 | acyl-CoA thioesterase 13 |
| ACOT2 | acyl-CoA thioesterase 2 |
| ACOT7 | acyl-CoA thioesterase 7 |
| ACOT9 | acyl-CoA thioesterase 9 |
| ACP6 | acid phosphatase 6, lysophosphatidic |
| ACSF2 | acyl-CoA synthetase family member 2 |
| ACSF3 | acyl-CoA synthetase family member 3 |
| ACSL1 | acyl-CoA synthetase long-chain family member 1 |
| ACSL3 | acyl-CoA synthetase long-chain family member 3 |
| ACSL4 | acyl-CoA synthetase long-chain family member 4 |
| ACSL5 | acyl-CoA synthetase long-chain family member 5 |
| ACSL6 | acyl-CoA synthetase long chain family member 6 |
| ACSM1 | acyl-CoA synthetase medium-chain family member 1 |
| ACSM2A | acyl-CoA synthetase medium-chain family member 2A |
| ACSM2B | acyl-CoA synthetase medium-chain family member 2B |
| ACSM3 | acyl-CoA synthetase medium-chain family member 3 |
| ACSM4 | acyl-CoA synthetase medium-chain family member 4 |
| ACSM5 | acyl-CoA synthetase medium-chain family member 5 |
| ACSM6 | acyl-CoA synthetase medium-chain family member 6 |
| ACSS1 | acyl-CoA synthetase short-chain family member 1 |
| ACSS3 | acyl-CoA synthetase short-chain family member 3 |
| ACTB | actin, beta |
| ADCK1 | aarF domain containing kinase 1 |
| ADCK2 | aarF domain containing kinase 2 |
| ADCK3 | aarF domain containing kinase 3 |
| ADCK4 | aarF domain containing kinase 4 |
| ADCK5 | aarF domain containing kinase 5 |
| ADCY10 | adenylate cyclase 10 (soluble) |
| ADHFE1 | alcohol dehydrogenase, iron containing, 1 |
| ADO | 2-aminoethanethiol (cysteamine) dioxygenase |
| ADPRHL2 | ADP-ribosylhydrolase like 2 |
| ADSS | adenylosuccinate synthase |
| AFG1L | AFG1 like ATPase |
| AFG3L2 | AFG3-like AAA ATPase 2 |
| AGK | acylglycerol kinase |
| AGMAT | agmatine ureohydrolase (agmatinase) |
| AGPAT4 | 1-acylglycerol-3-phosphate O-acyltransferase 4 |
| AGPAT5 | 1-acylglycerol-3-phosphate O-acyltransferase 5 |
| AGXT | alanine--glyoxylate and serine--pyruvate aminotransferase |
| AGXT2 | alanine--glyoxylate aminotransferase 2 |
| AHCYL1 | adenosylhomocysteinase like 1 |
| AIFM1 | apoptosis-inducing factor, mitochondrion-associated, 1 |
| AIFM2 | apoptosis-inducing factor, mitochondrion-associated, 2 |
| AIFM3 | apoptosis-inducing factor, mitochondrion-associated, 3 |
| AK2 | adenylate kinase 2 |
| AK3 | adenylate kinase 3 |
| AK4 | adenylate kinase 4 |
| AKAP1 | A kinase (PRKA) anchor protein 1 |
| AKAP10 | A kinase (PRKA) anchor protein 10 |
| AKR1B10 | aldo-keto reductase family 1 member B10 |
| AKR1B15 | aldo-keto reductase family 1, member B15 |
| AKR7A2 | aldo-keto reductase family 7, member A2 |
| AKT1 | v-akt murine thymoma viral oncogene homolog 1 |
| ALAS1 | 5-aminolevulinate synthase 1 |
| ALAS2 | 5-aminolevulinate synthase 2 |
| ALDH18A1 | aldehyde dehydrogenase 18 family, member A1 |
| ALDH1B1 | aldehyde dehydrogenase 1 family, member B1 |
| ALDH1L1 | aldehyde dehydrogenase 1 family, member L1 |
| ALDH1L2 | aldehyde dehydrogenase 1 family, member L2 |
| ALDH2 | aldehyde dehydrogenase 2 family (mitochondrial) |
| ALDH3A2 | aldehyde dehydrogenase 3 family, member A2 |
| ALDH4A1 | aldehyde dehydrogenase 4 family, member A1 |
| ALDH5A1 | aldehyde dehydrogenase 5 family, member A1 |
| ALDH6A1 | aldehyde dehydrogenase 6 family, member A1 |
| ALDH7A1 | aldehyde dehydrogenase 7 family, member A1 |
| ALDH9A1 | aldehyde dehydrogenase 9 family, member A1 |
| ALKBH1 | alkB homolog 1, histone H2A dioxygenase |
| ALKBH7 | alkB homolog 7 |
| AMACR | alpha-methylacyl-CoA racemase |
| AMBRA1 | autophagy/beclin-1 regulator 1 |
| AMT | aminomethyltransferase |
| ANGEL2 | angel homolog 2 (Drosophila) |
| ANTKMT | adenine nucleotide translocase lysine methyltransferase |
| ANXA6 | annexin A6 |
| APEX1 | APEX nuclease (multifunctional DNA repair enzyme) 1 |
| APEX2 | APEX nuclease (apurinic/apyrimidinic endonuclease) 2 |
| APOA1BP | apolipoprotein A-I binding protein |
| APOO | apolipoprotein O |
| APOOL | apolipoprotein O-like |
| APOPT1 | apoptogenic 1, mitochondrial |
| APTX | aprataxin |
| AR | androgen receptor |
| ARF5 | ADP ribosylation factor 5 |
| ARG2 | arginase 2 |
| ARL2 | ADP-ribosylation factor-like 2 |
| ARL2BP | ADP-ribosylation factor-like 2 binding protein |
| ARMC1 | armadillo repeat containing 1 |
| ARMC10 | armadillo repeat containing 10 |
| ARMCX1 | armadillo repeat containing, X-linked 1 |
| ARMCX2 | armadillo repeat containing, X-linked 2 |
| ARMCX3 | armadillo repeat containing, X-linked 3 |
| ARMCX6 | armadillo repeat containing X-linked 6 |
| ARMS2 | age-related maculopathy susceptibility 2 |
| ASAH2 | N-acylsphingosine amidohydrolase (non-lysosomal ceramidase) 2 |
| ASB9 | ankyrin repeat and SOCS box containing 9 |
| ATAD1 | ATPase family, AAA domain containing 1 |
| ATAD3A | ATPase family, AAA domain containing 3A |
| ATAD3B | ATPase family, AAA domain containing 3B |
| ATCAY | ataxia, cerebellar, Cayman type |
| ATG2A | autophagy related 2A |
| ATG4D | autophagy related 4D, cysteine peptidase |
| ATIC | 5-aminoimidazole-4-carboxamide ribonucleotide formyltransferase/IMP cyclohydrolase |
| ATP23 | ATP23 metallopeptidase and ATP synthase assembly factor homolog |
| ATP5A1 | ATP synthase, H+ transporting, mitochondrial F1 complex, alpha subunit 1, cardiac muscle |
| ATP5B | ATP synthase, H+ transporting, mitochondrial F1 complex, beta polypeptide |
| ATP5C1 | ATP synthase, H+ transporting, mitochondrial F1 complex, gamma polypeptide 1 |
| ATP5D | ATP synthase, H+ transporting, mitochondrial F1 complex, delta subunit |
| ATP5E | ATP synthase, H+ transporting, mitochondrial F1 complex, epsilon subunit |
| ATP5EP2 | ATP synthase, H+ transporting, mitochondrial F1 complex, epsilon subunit pseudogene 2 |
| ATP5F1 | ATP synthase, H+ transporting, mitochondrial Fo complex, subunit B1 |
| ATP5F1A | ATP synthase F1 subunit alpha |
| ATP5F1B | ATP synthase F1 subunit beta |
| ATP5F1C | ATP synthase F1 subunit gamma |
| ATP5F1D | ATP synthase F1 subunit delta |
| ATP5F1E | ATP synthase F1 subunit epsilon |
| ATP5G1 | ATP synthase, H+ transporting, mitochondrial Fo complex, subunit C1 (subunit 9) |
| ATP5G2 | ATP synthase, H+ transporting, mitochondrial Fo complex, subunit C2 (subunit 9) |
| ATP5G3 | ATP synthase, H+ transporting, mitochondrial Fo complex, subunit C3 (subunit 9) |
| ATP5H | ATP synthase, H+ transporting, mitochondrial Fo complex, subunit d |
| ATP5I | ATP synthase, H+ transporting, mitochondrial Fo complex, subunit E |
| ATP5IF1 | ATP synthase inhibitory factor subunit 1 |
| ATP5J | ATP synthase, H+ transporting, mitochondrial Fo complex, subunit F6 |
| ATP5J2 | ATP synthase, H+ transporting, mitochondrial Fo complex, subunit F2 |
| ATP5L | ATP synthase, H+ transporting, mitochondrial Fo complex, subunit G |
| ATP5L2 | ATP synthase, H+ transporting, mitochondrial Fo complex, subunit G2 |
| ATP5MC1 | ATP synthase membrane subunit c locus 1 |
| ATP5MC2 | ATP synthase membrane subunit c locus 2 |
| ATP5MC3 | ATP synthase membrane subunit c locus 3 |
| ATP5MD | ATP synthase membrane subunit DAPIT |
| ATP5ME | ATP synthase membrane subunit e |
| ATP5MF | ATP synthase membrane subunit f |
| ATP5MF-PTCD1 | ATP5MF-PTCD1 readthrough |
| ATP5MG | ATP synthase membrane subunit g |
| ATP5MPL | ATP synthase membrane subunit 6.8PL |
| ATP5O | ATP synthase, H+ transporting, mitochondrial F1 complex, O subunit |
| ATP5PB | ATP synthase peripheral stalk-membrane subunit b |
| ATP5PD | ATP synthase peripheral stalk subunit d |
| ATP5PF | ATP synthase peripheral stalk subunit F6 |
| ATP5PO | ATP synthase peripheral stalk subunit OSCP |
| ATP5S | ATP synthase, H+ transporting, mitochondrial Fo complex, subunit s (factor B) |
| ATP5SL | ATP5S-like |
| ATP7B | ATPase, Cu++ transporting, beta polypeptide |
| ATPAF1 | ATP synthase mitochondrial F1 complex assembly factor 1 |
| ATPAF2 | ATP synthase mitochondrial F1 complex assembly factor 2 |
| ATPIF1 | ATPase inhibitory factor 1 |
| ATPSCKMT | ATP synthase c subunit lysine N-methyltransferase |
| AUH | AU RNA binding protein/enoyl-CoA hydratase |
| AURKA | aurora kinase A |
| AURKAIP1 | aurora kinase A interacting protein 1 |
| BAD | BCL2-associated agonist of cell death |
| BAG2 | BCL2-associated athanogene 2 |
| BAG5 | BCL2-associated athanogene 5 |
| BAK1 | BCL2-antagonist/killer 1 |
| BAX | BCL2-associated X protein |
| BBC3 | BCL2 binding component 3 |
| BCAT1 | branched chain amino-acid transaminase 1, cytosolic |
| BCAT2 | branched chain amino-acid transaminase 2, mitochondrial |
| BCKDHA | branched chain keto acid dehydrogenase E1, alpha polypeptide |
| BCKDHB | branched chain keto acid dehydrogenase E1, beta polypeptide |
| BCKDK | branched chain ketoacid dehydrogenase kinase |
| BCL2 | B-cell CLL/lymphoma 2 |
| BCL2A1 | BCL2 related protein A1 |
| BCL2L1 | BCL2-like 1 |
| BCL2L10 | BCL2 like 10 |
| BCL2L11 | BCL2-like 11 (apoptosis facilitator) |
| BCL2L13 | BCL2-like 13 (apoptosis facilitator) |
| BCL2L2 | BCL2 like 2 |
| BCO2 | beta-carotene oxygenase 2 |
| BCS1L | BC1 (ubiquinol-cytochrome c reductase) synthesis-like |
| BDH1 | 3-hydroxybutyrate dehydrogenase, type 1 |
| BID | BH3 interacting domain death agonist |
| BIK | BCL2 interacting killer |
| BLID | BH3-like motif containing, cell death inducer |
| BLOC1S1 | biogenesis of lysosomal organelles complex-1, subunit 1 |
| BLOC1S2 | biogenesis of lysosomal organelles complex-1, subunit 2 |
| BNIP1 | BCL2/adenovirus E1B 19kDa interacting protein 1 |
| BNIP3 | BCL2/adenovirus E1B 19kDa interacting protein 3 |
| BNIP3L | BCL2/adenovirus E1B 19kDa interacting protein 3-like |
| BOK | BCL2 family apoptosis regulator BOK |
| BOLA1 | bolA family member 1 |
| BOLA3 | bolA family member 3 |
| BPHL | biphenyl hydrolase like |
| BRCA1 | breast cancer 1, early onset |
| BRINP3 | bone morphogenetic protein/retinoic acid inducible neural-specific 3 |
| BZRAP1 | benzodiazepine receptor (peripheral) associated protein 1 |
| C10orf10 | chromosome 10 open reading frame 10 |
| C10orf2 | chromosome 10 open reading frame 2 |
| C12orf10 | chromosome 12 open reading frame 10 |
| C12orf65 | chromosome 12 open reading frame 65 |
| C12orf73 | chromosome 12 open reading frame 73 |
| C14orf159 | chromosome 14 open reading frame 159 |
| C14orf2 | chromosome 14 open reading frame 2 |
| C15orf48 | chromosome 15 open reading frame 48 |
| C15orf61 | chromosome 15 open reading frame 61 |
| C15orf62 | chromosome 15 open reading frame 62 |
| C16orf91 | chromosome 16 open reading frame 91 |
| C17orf89 | chromosome 17 open reading frame 89 |
| C19orf12 | chromosome 19 open reading frame 12 |
| C19orf52 | chromosome 19 open reading frame 52 |
| C19orf70 | chromosome 19 open reading frame 70 |
| C1QBP | complement component 1, q subcomponent binding protein |
| C21orf33 | chromosome 21 open reading frame 33 |
| C2orf47 | chromosome 2 open reading frame 47 |
| C2orf69 | chromosome 2 open reading frame 69 |
| C3orf33 | chromosome 3 open reading frame 33 |
| C5orf63 | chromosome 5 open reading frame 63 |
| C6orf136 | chromosome 6 open reading frame 136 |
| C6orf203 | chromosome 6 open reading frame 203 |
| C7orf55 | chromosome 7 open reading frame 55 |
| C8orf82 | chromosome 8 open reading frame 82 |
| C9orf89 | chromosome 9 open reading frame 89 |
| CA5A | carbonic anhydrase VA, mitochondrial |
| CA5B | carbonic anhydrase VB, mitochondrial |
| CABS1 | calcium-binding protein, spermatid-specific 1 |
| CALR | calreticulin |
| CANX | calnexin |
| CAPN10 | calpain 10 |
| CAPRIN2 | caprin family member 2 |
| CARKD | carbohydrate kinase domain containing |
| CARS2 | cysteinyl-tRNA synthetase 2, mitochondrial (putative) |
| CASP2 | caspase 2, apoptosis-related cysteine peptidase |
| CASP3 | caspase 3 |
| CASP8 | caspase 8 |
| CASP9 | caspase 9 |
| CAT | catalase |
| CBR3 | carbonyl reductase 3 |
| CBR4 | carbonyl reductase 4 |
| CBS | cystathionine-beta-synthase |
| CCAR2 | cell cycle and apoptosis regulator 2 |
| CCBL2 | cysteine conjugate-beta lyase 2 |
| CCDC109B | coiled-coil domain containing 109B |
| CCDC127 | coiled-coil domain containing 127 |
| CCDC51 | coiled-coil domain containing 51 |
| CCDC58 | coiled-coil domain containing 58 |
| CCDC90B | coiled-coil domain containing 90B |
| CCS | copper chaperone for superoxide dismutase |
| CD3EAP | CD3e molecule, epsilon associated protein |
| CDC25C | cell division cycle 25C |
| CDK1 | cyclin-dependent kinase 1 |
| CDK5RAP1 | CDK5 regulatory subunit associated protein 1 |
| CEBPZOS | CEBPZ opposite strand |
| CECR5 | cat eye syndrome chromosome region, candidate 5 |
| CEND1 | cell cycle exit and neuronal differentiation 1 |
| CEP89 | centrosomal protein 89kDa |
| CFL1 | cofilin 1 (non-muscle) |
| CHCHD1 | coiled-coil-helix-coiled-coil-helix domain containing 1 |
| CHCHD10 | coiled-coil-helix-coiled-coil-helix domain containing 10 |
| CHCHD2 | coiled-coil-helix-coiled-coil-helix domain containing 2 |
| CHCHD3 | coiled-coil-helix-coiled-coil-helix domain containing 3 |
| CHCHD4 | coiled-coil-helix-coiled-coil-helix domain containing 4 |
| CHCHD5 | coiled-coil-helix-coiled-coil-helix domain containing 5 |
| CHCHD6 | coiled-coil-helix-coiled-coil-helix domain containing 6 |
| CHCHD7 | coiled-coil-helix-coiled-coil-helix domain containing 7 |
| CHDH | choline dehydrogenase |
| CHPT1 | choline phosphotransferase 1 |
| CIAPIN1 | cytokine induced apoptosis inhibitor 1 |
| CISD1 | CDGSH iron sulfur domain 1 |
| CISD2 | CDGSH iron sulfur domain 2 |
| CISD3 | CDGSH iron sulfur domain 3 |
| CKMT1A | creatine kinase, mitochondrial 1A |
| CKMT1B | creatine kinase, mitochondrial 1B |
| CKMT2 | creatine kinase, mitochondrial 2 (sarcomeric) |
| CLIC1 | chloride intracellular channel 1 |
| CLIC4 | chloride intracellular channel 4 |
| CLIC5 | chloride intracellular channel 5 |
| CLPB | ClpB homolog, mitochondrial AAA ATPase chaperonin |
| CLPP | caseinolytic mitochondrial matrix peptidase proteolytic subunit |
| CLPX | caseinolytic mitochondrial matrix peptidase chaperone subunit |
| CLUH | clustered mitochondria (cluA/CLU1) homolog |
| CLYBL | citrate lyase beta like |
| CMC1 | C-x(9)-C motif containing 1 |
| CMC2 | C-x(9)-C motif containing 2 |
| CMC4 | C-X9-C motif containing 4 |
| CMPK2 | cytidine monophosphate (UMP-CMP) kinase 2, mitochondrial |
| CNP | 2,3-cyclic nucleotide 3 phosphodiesterase |
| COA1 | cytochrome c oxidase assembly factor 1 homolog |
| COA3 | cytochrome c oxidase assembly factor 3 |
| COA4 | cytochrome c oxidase assembly factor 4 homolog |
| COA5 | cytochrome c oxidase assembly factor 5 |
| COA6 | cytochrome c oxidase assembly factor 6 |
| COA7 | cytochrome c oxidase assembly factor 7 (putative) |
| COA8 | cytochrome c oxidase assembly factor 8 |
| COASY | CoA synthase |
| COMT | catechol-O-methyltransferase |
| COMTD1 | catechol-O-methyltransferase domain containing 1 |
| COQ10A | coenzyme Q10A |
| COQ10B | coenzyme Q10B |
| COQ2 | coenzyme Q2 4-hydroxybenzoate polyprenyltransferase |
| COQ3 | coenzyme Q3 methyltransferase |
| COQ4 | coenzyme Q4 |
| COQ5 | coenzyme Q5, methyltransferase |
| COQ6 | coenzyme Q6 monooxygenase |
| COQ7 | coenzyme Q7 homolog, ubiquinone (yeast) |
| COQ8A | coenzyme Q8A |
| COQ8B | coenzyme Q8B |
| COQ9 | coenzyme Q9 |
| COX10 | COX10 heme A:farnesyltransferase cytochrome c oxidase assembly factor |
| COX11 | COX11 cytochrome c oxidase copper chaperone |
| COX14 | COX14 cytochrome c oxidase assembly factor |
| COX15 | cytochrome c oxidase assembly homolog 15 (yeast) |
| COX16 | COX16 cytochrome c oxidase assembly homolog |
| COX17 | COX17 cytochrome c oxidase copper chaperone |
| COX18 | COX18 cytochrome c oxidase assembly factor |
| COX19 | COX19 cytochrome c oxidase assembly factor |
| COX20 | COX20 cytochrome c oxidase assembly factor |
| COX4I1 | cytochrome c oxidase subunit IV isoform 1 |
| COX4I2 | cytochrome c oxidase subunit IV isoform 2 (lung) |
| COX5A | cytochrome c oxidase subunit Va |
| COX5B | cytochrome c oxidase subunit Vb |
| COX6A1 | cytochrome c oxidase subunit VIa polypeptide 1 |
| COX6A2 | cytochrome c oxidase subunit VIa polypeptide 2 |
| COX6B1 | cytochrome c oxidase subunit VIb polypeptide 1 (ubiquitous) |
| COX6B2 | cytochrome c oxidase subunit VIb polypeptide 2 (testis) |
| COX6C | cytochrome c oxidase subunit VIc |
| COX7A1 | cytochrome c oxidase subunit VIIa polypeptide 1 (muscle) |
| COX7A2 | cytochrome c oxidase subunit VIIa polypeptide 2 (liver) |
| COX7A2L | cytochrome c oxidase subunit VIIa polypeptide 2 like |
| COX7B | cytochrome c oxidase subunit VIIb |
| COX7B2 | cytochrome c oxidase subunit VIIb2 |
| COX7C | cytochrome c oxidase subunit VIIc |
| COX8A | cytochrome c oxidase subunit VIIIA (ubiquitous) |
| COX8C | cytochrome c oxidase subunit VIIIC |
| CPOX | coproporphyrinogen oxidase |
| CPS1 | carbamoyl-phosphate synthase 1, mitochondrial |
| CPT1A | carnitine palmitoyltransferase 1A (liver) |
| CPT1B | carnitine palmitoyltransferase 1B (muscle) |
| CPT1C | carnitine palmitoyltransferase 1C |
| CPT2 | carnitine palmitoyltransferase 2 |
| CRAT | carnitine O-acetyltransferase |
| CREB1 | cAMP responsive element binding protein 1 |
| CRLS1 | cardiolipin synthase 1 |
| CROT | carnitine O-octanoyltransferase |
| CRY1 | cryptochrome circadian clock 1 |
| CRYZ | crystallin, zeta (quinone reductase) |
| CS | citrate synthase |
| CSKMT | citrate synthase lysine methyltransferase |
| CUL2 | cullin 2 |
| CUTA | cutA divalent cation tolerance homolog (E. coli) |
| CYB5B | cytochrome b5 type B (outer mitochondrial membrane) |
| CYB5R3 | cytochrome b5 reductase 3 |
| CYC1 | cytochrome c-1 |
| CYCS | cytochrome c, somatic |
| CYP11A1 | cytochrome P450, family 11, subfamily A, polypeptide 1 |
| CYP11B1 | cytochrome P450, family 11, subfamily B, polypeptide 1 |
| CYP11B2 | cytochrome P450, family 11, subfamily B, polypeptide 2 |
| CYP1A1 | cytochrome P450, family 1, subfamily A, polypeptide 1 |
| CYP24A1 | cytochrome P450, family 24, subfamily A, polypeptide 1 |
| CYP27A1 | cytochrome P450, family 27, subfamily A, polypeptide 1 |
| CYP27B1 | cytochrome P450, family 27, subfamily B, polypeptide 1 |
| D2HGDH | D-2-hydroxyglutarate dehydrogenase |
| DAOA | D-amino acid oxidase activator |
| DAP3 | death associated protein 3 |
| DARS2 | aspartyl-tRNA synthetase 2, mitochondrial |
| DBI | diazepam binding inhibitor (GABA receptor modulator, acyl-CoA binding protein) |
| DBT | dihydrolipoamide branched chain transacylase E2 |
| DCAKD | dephospho-CoA kinase domain containing |
| DCXR | dicarbonyl/L-xylulose reductase |
| DDIT4 | DNA-damage-inducible transcript 4 |
| DDX28 | DEAD (Asp-Glu-Ala-Asp) box polypeptide 28 |
| DDX3X | DEAD (Asp-Glu-Ala-Asp) box helicase 3, X-linked |
| DECR1 | 2,4-dienoyl CoA reductase 1, mitochondrial |
| DELE1 | DAP3 binding cell death enhancer 1 |
| DGLUCY | D-glutamate cyclase |
| DGUOK | deoxyguanosine kinase |
| DHFRL1 | dihydrofolate reductase-like 1 |
| DHODH | dihydroorotate dehydrogenase (quinone) |
| DHRS1 | dehydrogenase/reductase 1 |
| DHRS2 | dehydrogenase/reductase (SDR family) member 2 |
| DHRS4 | dehydrogenase/reductase (SDR family) member 4 |
| DHRS7B | dehydrogenase/reductase 7B |
| DHTKD1 | dehydrogenase E1 and transketolase domain containing 1 |
| DHX30 | DEAH (Asp-Glu-Ala-His) box helicase 30 |
| DHX32 | DEAH (Asp-Glu-Ala-His) box polypeptide 32 |
| DIABLO | diablo, IAP-binding mitochondrial protein |
| DISC1 | disrupted in schizophrenia 1 |
| DLAT | dihydrolipoamide S-acetyltransferase |
| DLD | dihydrolipoamide dehydrogenase |
| DLST | dihydrolipoamide S-succinyltransferase (E2 component of 2-oxo-glutarate complex) |
| DMAC1 | distal membrane arm assembly complex 1 |
| DMAC2 | distal membrane arm assembly complex 2 |
| DMAC2L | distal membrane arm assembly complex 2 like |
| DMGDH | dimethylglycine dehydrogenase |
| DMPK | dystrophia myotonica-protein kinase |
| DNA2 | DNA replication helicase/nuclease 2 |
| DNAJA3 | DnaJ (Hsp40) homolog, subfamily A, member 3 |
| DNAJC11 | DnaJ (Hsp40) homolog, subfamily C, member 11 |
| DNAJC15 | DnaJ (Hsp40) homolog, subfamily C, member 15 |
| DNAJC19 | DnaJ (Hsp40) homolog, subfamily C, member 19 |
| DNAJC28 | DnaJ heat shock protein family (Hsp40) member C28 |
| DNAJC30 | DnaJ (Hsp40) homolog, subfamily C, member 30 |
| DNAJC4 | DnaJ heat shock protein family (Hsp40) member C4 |
| DNLZ | DNL-type zinc finger |
| DNM1L | dynamin 1-like |
| DNMT1 | DNA (cytosine-5-)-methyltransferase 1 |
| DNMT3A | DNA (cytosine-5-)-methyltransferase 3 alpha |
| DOK4 | docking protein 4 |
| DPYSL4 | dihydropyrimidinase-like 4 |
| DTYMK | deoxythymidylate kinase (thymidylate kinase) |
| DUS2 | dihydrouridine synthase 2 |
| DUSP18 | dual specificity phosphatase 18 |
| DUSP21 | dual specificity phosphatase 21 |
| DUT | deoxyuridine triphosphatase |
| EARS2 | glutamyl-tRNA synthetase 2, mitochondrial |
| ECH1 | enoyl CoA hydratase 1, peroxisomal |
| ECHDC1 | ethylmalonyl-CoA decarboxylase 1 |
| ECHDC2 | enoyl CoA hydratase domain containing 2 |
| ECHDC3 | enoyl CoA hydratase domain containing 3 |
| ECHS1 | enoyl CoA hydratase, short chain, 1, mitochondrial |
| ECI1 | enoyl-CoA delta isomerase 1 |
| ECI2 | enoyl-CoA delta isomerase 2 |
| ECSIT | ECSIT signalling integrator |
| EFCAB6 | EF-hand calcium binding domain 6 |
| EFHD1 | EF-hand domain family, member D1 |
| EHHADH | enoyl-CoA hydratase and 3-hydroxyacyl CoA dehydrogenase |
| EIF5A | eukaryotic translation initiation factor 5A |
| ELAC2 | elaC ribonuclease Z 2 |
| EMC8 | ER membrane protein complex subunit 8 |
| ENDOG | endonuclease G |
| ENO1 | enolase 1, (alpha) |
| ENOSF1 | enolase superfamily member 1 |
| EPHX2 | epoxide hydrolase 2 |
| ERAL1 | Era-like 12S mitochondrial rRNA chaperone 1 |
| ERCC6L2 | excision repair cross-complementation group 6-like 2 |
| ERCC8 | excision repair cross-complementation group 8 |
| ESR2 | estrogen receptor 2 (ER beta) |
| ETFA | electron-transfer-flavoprotein, alpha polypeptide |
| ETFB | electron-transfer-flavoprotein, beta polypeptide |
| ETFBKMT | electron transfer flavoprotein subunit beta lysine methyltransferase |
| ETFDH | electron-transferring-flavoprotein dehydrogenase |
| ETFRF1 | electron transfer flavoprotein regulatory factor 1 |
| ETHE1 | ethylmalonic encephalopathy 1 |
| EXD2 | exonuclease 3-5 domain containing 2 |
| EXOG | endo/exonuclease (5-3), endonuclease G-like |
| FABP1 | fatty acid binding protein 1 |
| FADS1 | fatty acid desaturase 1 |
| FAHD1 | fumarylacetoacetate hydrolase domain containing 1 |
| FAHD2A | fumarylacetoacetate hydrolase domain containing 2A |
| FAHD2B | fumarylacetoacetate hydrolase domain containing 2B |
| FAM136A | family with sequence similarity 136, member A |
| FAM162A | family with sequence similarity 162, member A |
| FAM173A | family with sequence similarity 173, member A |
| FAM173B | family with sequence similarity 173, member B |
| FAM185A | family with sequence similarity 185, member A |
| FAM210A | family with sequence similarity 210, member A |
| FAM210B | family with sequence similarity 210, member B |
| FAM73A | family with sequence similarity 73, member A |
| FAM73B | family with sequence similarity 73, member B |
| FAM92A1 | family with sequence similarity 92, member A1 |
| FANCG | Fanconi anemia, complementation group G |
| FARS2 | phenylalanyl-tRNA synthetase 2, mitochondrial |
| FASN | fatty acid synthase |
| FASTK | Fas-activated serine/threonine kinase |
| FASTKD1 | FAST kinase domains 1 |
| FASTKD2 | FAST kinase domains 2 |
| FASTKD3 | FAST kinase domains 3 |
| FASTKD5 | FAST kinase domains 5 |
| FBXL4 | F-box and leucine-rich repeat protein 4 |
| FBXO17 | F-box protein 17 |
| FBXO7 | F-box protein 7 |
| FDPS | farnesyl diphosphate synthase |
| FDX1 | ferredoxin 1 |
| FDX1L | ferredoxin 1-like |
| FDX2 | ferredoxin 2 |
| FDXR | ferredoxin reductase |
| FECH | ferrochelatase |
| FEN1 | flap structure-specific endonuclease 1 |
| FH | fumarate hydratase |
| FHIT | fragile histidine triad |
| FHL1 | four and a half LIM domains 1 |
| FIS1 | fission, mitochondrial 1 |
| FKBP10 | FK506 binding protein 10, 65 kDa |
| FKBP4 | FK506 binding protein 4, 59kDa |
| FKBP8 | FK506 binding protein 8, 38kDa |
| FLAD1 | flavin adenine dinucleotide synthetase 1 |
| FLVCR1 | feline leukemia virus subgroup C cellular receptor 1 |
| FMC1 | formation of mitochondrial complex V assembly factor 1 homolog |
| FOXM1 | forkhead box M1 |
| FOXRED1 | FAD-dependent oxidoreductase domain containing 1 |
| FPGS | folylpolyglutamate synthase |
| FTH1 | ferritin heavy chain 1 |
| FTMT | ferritin mitochondrial |
| FTSJ2 | FtsJ RNA methyltransferase homolog 2 (E. coli) |
| FUNDC1 | FUN14 domain containing 1 |
| FUNDC2 | FUN14 domain containing 2 |
| FXN | frataxin |
| FYN | FYN proto-oncogene, Src family tyrosine kinase |
| G0S2 | G0/G1 switch 2 |
| GADD45GIP1 | growth arrest and DNA-damage-inducible, gamma interacting protein 1 |
| GAPDH | glyceraldehyde-3-phosphate dehydrogenase |
| GARS | glycyl-tRNA synthetase |
| GARS1 | glycyl-tRNA synthetase 1 |
| GATB | glutamyl-tRNA(Gln) amidotransferase, subunit B |
| GATC | glutamyl-tRNA(Gln) amidotransferase, subunit C |
| GATD3A | glutamine amidotransferase like class 1 domain containing 3A |
| GATM | glycine amidinotransferase (L-arginine:glycine amidinotransferase) |
| GBAS | glioblastoma amplified sequence |
| GCAT | glycine C-acetyltransferase |
| GCDH | glutaryl-CoA dehydrogenase |
| GCSH | glycine cleavage system protein H (aminomethyl carrier) |
| GDAP1 | ganglioside induced differentiation associated protein 1 |
| GDAP1L1 | ganglioside induced differentiation associated protein 1-like 1 |
| GDF5OS | growth differentiation factor 5 opposite strand |
| GFER | growth factor, augmenter of liver regeneration |
| GFM1 | G elongation factor, mitochondrial 1 |
| GFM2 | G elongation factor, mitochondrial 2 |
| GHITM | growth hormone inducible transmembrane protein |
| GJA1 | gap junction protein, alpha 1, 43kDa |
| GK | glycerol kinase |
| GK2 | glycerol kinase 2 |
| GLDC | glycine dehydrogenase (decarboxylating) |
| GLOD4 | glyoxalase domain containing 4 |
| GLRX2 | glutaredoxin 2 |
| GLRX5 | glutaredoxin 5 |
| GLS | glutaminase |
| GLS2 | glutaminase 2 (liver, mitochondrial) |
| GLUD1 | glutamate dehydrogenase 1 |
| GLUD2 | glutamate dehydrogenase 2 |
| GLYAT | glycine-N-acyltransferase |
| GLYCTK | glycerate kinase |
| GOLPH3 | golgi phosphoprotein 3 (coat-protein) |
| GOT2 | glutamic-oxaloacetic transaminase 2, mitochondrial |
| GPAM | glycerol-3-phosphate acyltransferase, mitochondrial |
| GPAT2 | glycerol-3-phosphate acyltransferase 2, mitochondrial |
| GPD1 | glycerol-3-phosphate dehydrogenase 1 (soluble) |
| GPD2 | glycerol-3-phosphate dehydrogenase 2 (mitochondrial) |
| GPS2 | G protein pathway suppressor 2 |
| GPT2 | glutamic pyruvate transaminase (alanine aminotransferase) 2 |
| GPX1 | glutathione peroxidase 1 |
| GPX4 | glutathione peroxidase 4 |
| GRAMD4 | GRAM domain containing 4 |
| GRHPR | glyoxylate reductase/hydroxypyruvate reductase |
| GRPEL1 | GrpE-like 1, mitochondrial (E. coli) |
| GRPEL2 | GrpE-like 2, mitochondrial (E. coli) |
| GRSF1 | G-rich RNA sequence binding factor 1 |
| GSR | glutathione reductase |
| GSTA4 | glutathione S-transferase alpha 4 |
| GSTK1 | glutathione S-transferase kappa 1 |
| GSTZ1 | glutathione S-transferase zeta 1 |
| GTPBP10 | GTP-binding protein 10 (putative) |
| GTPBP3 | GTP binding protein 3 (mitochondrial) |
| GTPBP6 | GTP binding protein 6 (putative) |
| GUF1 | GUF1 homolog, GTPase |
| GUK1 | guanylate kinase 1 |
| HADH | hydroxyacyl-CoA dehydrogenase |
| HADHA | hydroxyacyl-CoA dehydrogenase/3-ketoacyl-CoA thiolase/enoyl-CoA hydratase (trifunctional protein), alpha subunit |
| HADHB | hydroxyacyl-CoA dehydrogenase/3-ketoacyl-CoA thiolase/enoyl-CoA hydratase (trifunctional protein), beta subunit |
| HAGH | hydroxyacylglutathione hydrolase |
| HAO2 | hydroxyacid oxidase 2 |
| HARS2 | histidyl-tRNA synthetase 2, mitochondrial |
| HAX1 | HCLS1 associated protein X-1 |
| HCCS | holocytochrome c synthase |
| HDHD3 | haloacid dehalogenase-like hydrolase domain containing 3 |
| HDHD5 | haloacid dehalogenase like hydrolase domain containing 5 |
| HEBP1 | heme binding protein 1 |
| HEMK1 | HemK methyltransferase family member 1 |
| HIBADH | 3-hydroxyisobutyrate dehydrogenase |
| HIBCH | 3-hydroxyisobutyryl-CoA hydrolase |
| HIGD1A | HIG1 hypoxia inducible domain family, member 1A |
| HIGD2A | HIG1 hypoxia inducible domain family, member 2A |
| HINT1 | histidine triad nucleotide binding protein 1 |
| HINT2 | histidine triad nucleotide binding protein 2 |
| HINT3 | histidine triad nucleotide binding protein 3 |
| HK1 | hexokinase 1 |
| HK2 | hexokinase 2 |
| HKDC1 | hexokinase domain containing 1 |
| HMGCL | 3-hydroxymethyl-3-methylglutaryl-CoA lyase |
| HMGCS2 | 3-hydroxy-3-methylglutaryl-CoA synthase 2 (mitochondrial) |
| HNRNPK | heterogeneous nuclear ribonucleoprotein K |
| HOGA1 | 4-hydroxy-2-oxoglutarate aldolase 1 |
| HPDL | 4-hydroxyphenylpyruvate dioxygenase-like |
| HRK | harakiri, BCL2 interacting protein |
| HRSP12 | heat-responsive protein 12 |
| HSCB | HscB mitochondrial iron-sulfur cluster co-chaperone |
| HSD17B10 | hydroxysteroid (17-beta) dehydrogenase 10 |
| HSD17B4 | hydroxysteroid 17-beta dehydrogenase 4 |
| HSD17B8 | hydroxysteroid (17-beta) dehydrogenase 8 |
| HSD3B1 | hydroxy-delta-5-steroid dehydrogenase, 3 beta- and steroid delta-isomerase 1 |
| HSDL1 | hydroxysteroid dehydrogenase like 1 |
| HSDL2 | hydroxysteroid dehydrogenase like 2 |
| HSPA1A | heat shock 70kDa protein 1A |
| HSPA5 | heat shock 70kDa protein 5 (glucose-regulated protein, 78kDa) |
| HSPA9 | heat shock 70kDa protein 9 (mortalin) |
| HSPB1 | heat shock 27kDa protein 1 |
| HSPD1 | heat shock 60kDa protein 1 (chaperonin) |
| HSPE1 | heat shock 10kDa protein 1 |
| HTATIP2 | HIV-1 Tat interactive protein 2 |
| HTD2 | hydroxyacyl-thioester dehydratase type 2 |
| HTRA2 | HtrA serine peptidase 2 |
| IARS2 | isoleucyl-tRNA synthetase 2, mitochondrial |
| IBA57 | IBA57 homolog, iron-sulfur cluster assembly |
| ICT1 | immature colon carcinoma transcript 1 |
| IDE | insulin-degrading enzyme |
| IDH2 | isocitrate dehydrogenase 2 (NADP+), mitochondrial |
| IDH3A | isocitrate dehydrogenase 3 (NAD+) alpha |
| IDH3B | isocitrate dehydrogenase 3 (NAD+) beta |
| IDH3G | isocitrate dehydrogenase 3 (NAD+) gamma |
| IDI1 | isopentenyl-diphosphate delta isomerase 1 |
| IFI27 | interferon, alpha-inducible protein 27 |
| IFI6 | interferon, alpha-inducible protein 6 |
| IMMP1L | inner mitochondrial membrane peptidase subunit 1 |
| IMMP2L | inner mitochondrial membrane peptidase subunit 2 |
| IMMT | inner membrane protein, mitochondrial |
| IRG1 | immunoresponsive 1 homolog (mouse) |
| IRGM | immunity-related GTPase family, M |
| ISCA1 | iron-sulfur cluster assembly 1 |
| ISCA2 | iron-sulfur cluster assembly 2 |
| ISCU | iron-sulfur cluster assembly enzyme |
| ISOC2 | isochorismatase domain containing 2 |
| ITGA3 | integrin, alpha 3 (antigen CD49C, alpha 3 subunit of VLA-3 receptor) |
| IVD | isovaleryl-CoA dehydrogenase |
| KANSL2 | KAT8 regulatory NSL complex subunit 2 |
| KARS | lysyl-tRNA synthetase |
| KARS1 | lysyl-tRNA synthetase 1 |
| KCNJ1 | potassium channel, inwardly rectifying subfamily J, member 1 |
| KCNJ11 | potassium channel, inwardly rectifying subfamily J, member 11 |
| KCNMA1 | potassium channel, calcium activated large conductance subfamily M alpha, member 1 |
| KIAA0101 | KIAA0101 |
| KIAA0141 | KIAA0141 |
| KIAA0391 | KIAA0391 |
| KIF1B | kinesin family member 1B |
| KLK6 | kallikrein-related peptidase 6 |
| KMO | kynurenine 3-monooxygenase (kynurenine 3-hydroxylase) |
| KYAT3 | kynurenine aminotransferase 3 |
| L2HGDH | L-2-hydroxyglutarate dehydrogenase |
| LACE1 | lactation elevated 1 |
| LACTB | lactamase, beta |
| LACTB2 | lactamase, beta 2 |
| LAMC1 | laminin, gamma 1 (formerly LAMB2) |
| LAP3 | leucine aminopeptidase 3 |
| LARP4 | La ribonucleoprotein domain family, member 4 |
| LARS2 | leucyl-tRNA synthetase 2, mitochondrial |
| LDHAL6B | lactate dehydrogenase A like 6B |
| LDHB | lactate dehydrogenase B |
| LDHD | lactate dehydrogenase D |
| LETM1 | leucine zipper-EF-hand containing transmembrane protein 1 |
| LETM2 | leucine zipper-EF-hand containing transmembrane protein 2 |
| LETMD1 | LETM1 domain containing 1 |
| LIAS | lipoic acid synthetase |
| LIG3 | ligase III, DNA, ATP-dependent |
| LIPT1 | lipoyltransferase 1 |
| LIPT2 | lipoyl(octanoyl) transferase 2 (putative) |
| LONP1 | lon peptidase 1, mitochondrial |
| LPCAT1 | lysophosphatidylcholine acyltransferase 1 |
| LRP2 | low density lipoprotein receptor-related protein 2 |
| LRPPRC | leucine-rich pentatricopeptide repeat containing |
| LRRC10 | leucine rich repeat containing 10 |
| LRRC59 | leucine rich repeat containing 59 |
| LRRK2 | leucine-rich repeat kinase 2 |
| LYPLA1 | lysophospholipase I |
| LYPLAL1 | lysophospholipase like 1 |
| LYRM1 | LYR motif containing 1 |
| LYRM2 | LYR motif containing 2 |
| LYRM4 | LYR motif containing 4 |
| LYRM7 | LYR motif containing 7 |
| LYRM9 | LYR motif containing 9 |
| MAATS1 | MYCBP-associated, testis expressed 1 |
| MACROD1 | MACRO domain containing 1 |
| MAIP1 | matrix AAA peptidase interacting protein 1 |
| MALSU1 | mitochondrial assembly of ribosomal large subunit 1 |
| MAOA | monoamine oxidase A |
| MAOB | monoamine oxidase B |
| MAPK12 | mitogen-activated protein kinase 12 |
| MARC1 | mitochondrial amidoxime reducing component 1 |
| MARC2 | mitochondrial amidoxime reducing component 2 |
| MARCH5 | membrane-associated ring finger (C3HC4) 5 |
| MARCHF5 | membrane associated ring-CH-type finger 5 |
| MARCKS | myristoylated alanine-rich protein kinase C substrate |
| MARS2 | methionyl-tRNA synthetase 2, mitochondrial |
| MARVELD1 | MARVEL domain containing 1 |
| MAVS | mitochondrial antiviral signaling protein |
| MCAT | malonyl CoA:ACP acyltransferase (mitochondrial) |
| MCCC1 | methylcrotonoyl-CoA carboxylase 1 (alpha) |
| MCCC2 | methylcrotonoyl-CoA carboxylase 2 (beta) |
| MCCD1 | mitochondrial coiled-coil domain 1 |
| MCEE | methylmalonyl CoA epimerase |
| MCL1 | myeloid cell leukemia 1 |
| MCRIP2 | MAPK regulated corepressor interacting protein 2 |
| MCU | mitochondrial calcium uniporter |
| MCUB | mitochondrial calcium uniporter dominant negative subunit beta |
| MCUR1 | mitochondrial calcium uniporter regulator 1 |
| MDH2 | malate dehydrogenase 2, NAD (mitochondrial) |
| ME2 | malic enzyme 2, NAD(+)-dependent, mitochondrial |
| ME3 | malic enzyme 3, NADP(+)-dependent, mitochondrial |
| MECR | mitochondrial trans-2-enoyl-CoA reductase |
| METAP1D | methionyl aminopeptidase type 1D (mitochondrial) |
| METTL12 | methyltransferase like 12 |
| METTL15 | methyltransferase like 15 |
| METTL17 | methyltransferase like 17 |
| METTL20 | methyltransferase like 20 |
| METTL4 | methyltransferase like 4 |
| METTL5 | methyltransferase like 5 |
| METTL8 | methyltransferase like 8 |
| MFF | mitochondrial fission factor |
| MFN1 | mitofusin 1 |
| MFN2 | mitofusin 2 |
| MGARP | mitochondria-localized glutamic acid-rich protein |
| MGME1 | mitochondrial genome maintenance exonuclease 1 |
| MGST1 | microsomal glutathione S-transferase 1 |
| MGST3 | microsomal glutathione S-transferase 3 |
| MICOS10 | mitochondrial contact site and cristae organizing system subunit 10 |
| MICOS13 | mitochondrial contact site and cristae organizing system subunit 13 |
| MICU1 | mitochondrial calcium uptake 1 |
| MICU2 | mitochondrial calcium uptake 2 |
| MICU3 | mitochondrial calcium uptake family, member 3 |
| MIEF1 | mitochondrial elongation factor 1 |
| MIEF2 | mitochondrial elongation factor 2 |
| MIGA1 | mitoguardin 1 |
| MIGA2 | mitoguardin 2 |
| MINOS1 | mitochondrial inner membrane organizing system 1 |
| MIPEP | mitochondrial intermediate peptidase |
| MLH1 | mutL homolog 1 |
| MLX | MLX, MAX dimerization protein |
| MLXIP | MLX interacting protein |
| MLYCD | malonyl-CoA decarboxylase |
| MMAA | methylmalonic aciduria (cobalamin deficiency) cblA type |
| MMAB | methylmalonic aciduria (cobalamin deficiency) cblB type |
| MMADHC | methylmalonic aciduria (cobalamin deficiency) cblD type, with homocystinuria |
| MMD | monocyte to macrophage differentiation-associated |
| MMUT | methylmalonyl-CoA mutase |
| MOBP | myelin-associated oligodendrocyte basic protein |
| MOCS1 | molybdenum cofactor synthesis 1 |
| MPC1 | mitochondrial pyruvate carrier 1 |
| MPC1L | mitochondrial pyruvate carrier 1 like |
| MPC2 | mitochondrial pyruvate carrier 2 |
| MPG | N-methylpurine DNA glycosylase |
| MPST | mercaptopyruvate sulfurtransferase |
| MPV17 | MpV17 mitochondrial inner membrane protein |
| MPV17L | MPV17 mitochondrial membrane protein-like |
| MPV17L2 | MPV17 mitochondrial membrane protein-like 2 |
| MRM1 | mitochondrial rRNA methyltransferase 1 |
| MRM2 | mitochondrial rRNA methyltransferase 2 |
| MRM3 | mitochondrial rRNA methyltransferase 3 |
| MRPL1 | mitochondrial ribosomal protein L1 |
| MRPL10 | mitochondrial ribosomal protein L10 |
| MRPL11 | mitochondrial ribosomal protein L11 |
| MRPL12 | mitochondrial ribosomal protein L12 |
| MRPL13 | mitochondrial ribosomal protein L13 |
| MRPL14 | mitochondrial ribosomal protein L14 |
| MRPL15 | mitochondrial ribosomal protein L15 |
| MRPL16 | mitochondrial ribosomal protein L16 |
| MRPL17 | mitochondrial ribosomal protein L17 |
| MRPL18 | mitochondrial ribosomal protein L18 |
| MRPL19 | mitochondrial ribosomal protein L19 |
| MRPL2 | mitochondrial ribosomal protein L2 |
| MRPL20 | mitochondrial ribosomal protein L20 |
| MRPL21 | mitochondrial ribosomal protein L21 |
| MRPL22 | mitochondrial ribosomal protein L22 |
| MRPL23 | mitochondrial ribosomal protein L23 |
| MRPL24 | mitochondrial ribosomal protein L24 |
| MRPL27 | mitochondrial ribosomal protein L27 |
| MRPL28 | mitochondrial ribosomal protein L28 |
| MRPL3 | mitochondrial ribosomal protein L3 |
| MRPL30 | mitochondrial ribosomal protein L30 |
| MRPL32 | mitochondrial ribosomal protein L32 |
| MRPL33 | mitochondrial ribosomal protein L33 |
| MRPL34 | mitochondrial ribosomal protein L34 |
| MRPL35 | mitochondrial ribosomal protein L35 |
| MRPL36 | mitochondrial ribosomal protein L36 |
| MRPL37 | mitochondrial ribosomal protein L37 |
| MRPL38 | mitochondrial ribosomal protein L38 |
| MRPL39 | mitochondrial ribosomal protein L39 |
| MRPL4 | mitochondrial ribosomal protein L4 |
| MRPL40 | mitochondrial ribosomal protein L40 |
| MRPL41 | mitochondrial ribosomal protein L41 |
| MRPL42 | mitochondrial ribosomal protein L42 |
| MRPL43 | mitochondrial ribosomal protein L43 |
| MRPL44 | mitochondrial ribosomal protein L44 |
| MRPL45 | mitochondrial ribosomal protein L45 |
| MRPL46 | mitochondrial ribosomal protein L46 |
| MRPL47 | mitochondrial ribosomal protein L47 |
| MRPL48 | mitochondrial ribosomal protein L48 |
| MRPL49 | mitochondrial ribosomal protein L49 |
| MRPL50 | mitochondrial ribosomal protein L50 |
| MRPL51 | mitochondrial ribosomal protein L51 |
| MRPL52 | mitochondrial ribosomal protein L52 |
| MRPL53 | mitochondrial ribosomal protein L53 |
| MRPL54 | mitochondrial ribosomal protein L54 |
| MRPL55 | mitochondrial ribosomal protein L55 |
| MRPL57 | mitochondrial ribosomal protein L57 |
| MRPL58 | mitochondrial ribosomal protein L58 |
| MRPL9 | mitochondrial ribosomal protein L9 |
| MRPS10 | mitochondrial ribosomal protein S10 |
| MRPS11 | mitochondrial ribosomal protein S11 |
| MRPS12 | mitochondrial ribosomal protein S12 |
| MRPS14 | mitochondrial ribosomal protein S14 |
| MRPS15 | mitochondrial ribosomal protein S15 |
| MRPS16 | mitochondrial ribosomal protein S16 |
| MRPS17 | mitochondrial ribosomal protein S17 |
| MRPS18A | mitochondrial ribosomal protein S18A |
| MRPS18B | mitochondrial ribosomal protein S18B |
| MRPS18C | mitochondrial ribosomal protein S18C |
| MRPS2 | mitochondrial ribosomal protein S2 |
| MRPS21 | mitochondrial ribosomal protein S21 |
| MRPS22 | mitochondrial ribosomal protein S22 |
| MRPS23 | mitochondrial ribosomal protein S23 |
| MRPS24 | mitochondrial ribosomal protein S24 |
| MRPS25 | mitochondrial ribosomal protein S25 |
| MRPS26 | mitochondrial ribosomal protein S26 |
| MRPS27 | mitochondrial ribosomal protein S27 |
| MRPS28 | mitochondrial ribosomal protein S28 |
| MRPS30 | mitochondrial ribosomal protein S30 |
| MRPS31 | mitochondrial ribosomal protein S31 |
| MRPS33 | mitochondrial ribosomal protein S33 |
| MRPS34 | mitochondrial ribosomal protein S34 |
| MRPS35 | mitochondrial ribosomal protein S35 |
| MRPS36 | mitochondrial ribosomal protein S36 |
| MRPS5 | mitochondrial ribosomal protein S5 |
| MRPS6 | mitochondrial ribosomal protein S6 |
| MRPS7 | mitochondrial ribosomal protein S7 |
| MRPS9 | mitochondrial ribosomal protein S9 |
| MRRF | mitochondrial ribosome recycling factor |
| MRS2 | MRS2 magnesium transporter |
| MSRA | methionine sulfoxide reductase A |
| MSRB2 | methionine sulfoxide reductase B2 |
| MSRB3 | methionine sulfoxide reductase B3 |
| MSS51 | MSS51 mitochondrial translational activator |
| MSTO1 | misato 1, mitochondrial distribution and morphology regulator |
| MTARC1 | mitochondrial amidoxime reducing component 1 |
| MTARC2 | mitochondrial amidoxime reducing component 2 |
| MT-ATP6 | mitochondrially encoded ATP synthase 6 |
| MT-ATP8 | mitochondrially encoded ATP synthase 8 |
| MTCH1 | mitochondrial carrier 1 |
| MTCH2 | mitochondrial carrier 2 |
| MT-CO1 | mitochondrially encoded cytochrome c oxidase I |
| MT-CO2 | mitochondrially encoded cytochrome c oxidase II |
| MT-CO3 | mitochondrially encoded cytochrome c oxidase III |
| MTCP1 | mature T-cell proliferation 1 |
| MT-CYB | mitochondrially encoded cytochrome b |
| MTERF1 | mitochondrial transcription termination factor 1 |
| MTERF2 | mitochondrial transcription termination factor 2 |
| MTERF3 | mitochondrial transcription termination factor 3 |
| MTERF4 | mitochondrial transcription termination factor 4 |
| MTFMT | mitochondrial methionyl-tRNA formyltransferase |
| MTFP1 | mitochondrial fission process 1 |
| MTFR1 | mitochondrial fission regulator 1 |
| MTFR1L | mitochondrial fission regulator 1-like |
| MTFR2 | mitochondrial fission regulator 2 |
| MTG1 | mitochondrial ribosome-associated GTPase 1 |
| MTG2 | mitochondrial ribosome-associated GTPase 2 |
| MTHFD1L | methylenetetrahydrofolate dehydrogenase (NADP+ dependent) 1-like |
| MTHFD2 | methylenetetrahydrofolate dehydrogenase (NADP+ dependent) 2, methenyltetrahydrofolate cyclohydrolase |
| MTHFD2L | methylenetetrahydrofolate dehydrogenase (NADP+ dependent) 2-like |
| MTHFS | 5,10-methenyltetrahydrofolate synthetase (5-formyltetrahydrofolate cyclo-ligase) |
| MTIF2 | mitochondrial translational initiation factor 2 |
| MTIF3 | mitochondrial translational initiation factor 3 |
| MT-ND1 | mitochondrially encoded NADH dehydrogenase 1 |
| MT-ND2 | mitochondrially encoded NADH dehydrogenase 2 |
| MT-ND3 | mitochondrially encoded NADH dehydrogenase 3 |
| MT-ND4 | mitochondrially encoded NADH dehydrogenase 4 |
| MT-ND4L | mitochondrially encoded NADH dehydrogenase 4L |
| MT-ND5 | mitochondrially encoded NADH dehydrogenase 5 |
| MT-ND6 | mitochondrially encoded NADH dehydrogenase 6 |
| MTO1 | mitochondrial tRNA translation optimization 1 |
| MTPAP | mitochondrial poly(A) polymerase |
| MTRES1 | mitochondrial transcription rescue factor 1 |
| MTRF1 | mitochondrial translational release factor 1 |
| MTRF1L | mitochondrial translational release factor 1-like |
| MTX1 | metaxin 1 |
| MTX2 | metaxin 2 |
| MTX3 | metaxin 3 |
| MUL1 | mitochondrial E3 ubiquitin protein ligase 1 |
| MUT | methylmalonyl CoA mutase |
| MUTYH | mutY DNA glycosylase |
| MYCBP | MYC binding protein |
| MYG1 | MYG1 exonuclease |
| MYH10 | myosin, heavy chain 10, non-muscle |
| MYH9 | myosin, heavy chain 9, non-muscle |
| MYO19 | myosin XIX |
| NADK2 | NAD kinase 2, mitochondrial |
| NAGS | N-acetylglutamate synthase |
| NAPG | N-ethylmaleimide-sensitive factor attachment protein, gamma |
| NARS2 | asparaginyl-tRNA synthetase 2, mitochondrial (putative) |
| NAT8L | N-acetyltransferase 8-like (GCN5-related, putative) |
| NAXD | NAD(P)HX dehydratase |
| NAXE | NAD(P)HX epimerase |
| NBR1 | neighbor of BRCA1 gene 1 |
| NDFIP2 | Nedd4 family interacting protein 2 |
| NDUFA1 | NADH dehydrogenase (ubiquinone) 1 alpha subcomplex, 1, 7.5kDa |
| NDUFA10 | NADH dehydrogenase (ubiquinone) 1 alpha subcomplex, 10, 42kDa |
| NDUFA11 | NADH dehydrogenase (ubiquinone) 1 alpha subcomplex, 11, 14.7kDa |
| NDUFA12 | NADH dehydrogenase (ubiquinone) 1 alpha subcomplex, 12 |
| NDUFA13 | NADH dehydrogenase (ubiquinone) 1 alpha subcomplex, 13 |
| NDUFA2 | NADH dehydrogenase (ubiquinone) 1 alpha subcomplex, 2, 8kDa |
| NDUFA3 | NADH dehydrogenase (ubiquinone) 1 alpha subcomplex, 3, 9kDa |
| NDUFA4 | NDUFA4, mitochondrial complex associated |
| NDUFA4L2 | NADH dehydrogenase (ubiquinone) 1 alpha subcomplex, 4-like 2 |
| NDUFA5 | NADH dehydrogenase (ubiquinone) 1 alpha subcomplex, 5 |
| NDUFA6 | NADH dehydrogenase (ubiquinone) 1 alpha subcomplex, 6, 14kDa |
| NDUFA7 | NADH dehydrogenase (ubiquinone) 1 alpha subcomplex, 7, 14.5kDa |
| NDUFA8 | NADH dehydrogenase (ubiquinone) 1 alpha subcomplex, 8, 19kDa |
| NDUFA9 | NADH dehydrogenase (ubiquinone) 1 alpha subcomplex, 9, 39kDa |
| NDUFAB1 | NADH dehydrogenase (ubiquinone) 1, alpha/beta subcomplex, 1, 8kDa |
| NDUFAF1 | NADH dehydrogenase (ubiquinone) complex I, assembly factor 1 |
| NDUFAF2 | NADH dehydrogenase (ubiquinone) complex I, assembly factor 2 |
| NDUFAF3 | NADH dehydrogenase (ubiquinone) complex I, assembly factor 3 |
| NDUFAF4 | NADH dehydrogenase (ubiquinone) complex I, assembly factor 4 |
| NDUFAF5 | NADH dehydrogenase (ubiquinone) complex I, assembly factor 5 |
| NDUFAF6 | NADH dehydrogenase (ubiquinone) complex I, assembly factor 6 |
| NDUFAF7 | NADH dehydrogenase (ubiquinone) complex I, assembly factor 7 |
| NDUFAF8 | NADH:ubiquinone oxidoreductase complex assembly factor 8 |
| NDUFB1 | NADH dehydrogenase (ubiquinone) 1 beta subcomplex, 1, 7kDa |
| NDUFB10 | NADH dehydrogenase (ubiquinone) 1 beta subcomplex, 10, 22kDa |
| NDUFB11 | NADH dehydrogenase (ubiquinone) 1 beta subcomplex, 11, 17.3kDa |
| NDUFB2 | NADH dehydrogenase (ubiquinone) 1 beta subcomplex, 2, 8kDa |
| NDUFB3 | NADH dehydrogenase (ubiquinone) 1 beta subcomplex, 3, 12kDa |
| NDUFB4 | NADH dehydrogenase (ubiquinone) 1 beta subcomplex, 4, 15kDa |
| NDUFB5 | NADH dehydrogenase (ubiquinone) 1 beta subcomplex, 5, 16kDa |
| NDUFB6 | NADH dehydrogenase (ubiquinone) 1 beta subcomplex, 6, 17kDa |
| NDUFB7 | NADH dehydrogenase (ubiquinone) 1 beta subcomplex, 7, 18kDa |
| NDUFB8 | NADH dehydrogenase (ubiquinone) 1 beta subcomplex, 8, 19kDa |
| NDUFB9 | NADH dehydrogenase (ubiquinone) 1 beta subcomplex, 9, 22kDa |
| NDUFC1 | NADH dehydrogenase (ubiquinone) 1, subcomplex unknown, 1, 6kDa |
| NDUFC2 | NADH dehydrogenase (ubiquinone) 1, subcomplex unknown, 2, 14.5kDa |
| NDUFS1 | NADH dehydrogenase (ubiquinone) Fe-S protein 1, 75kDa (NADH-coenzyme Q reductase) |
| NDUFS2 | NADH dehydrogenase (ubiquinone) Fe-S protein 2, 49kDa (NADH-coenzyme Q reductase) |
| NDUFS3 | NADH dehydrogenase (ubiquinone) Fe-S protein 3, 30kDa (NADH-coenzyme Q reductase) |
| NDUFS4 | NADH dehydrogenase (ubiquinone) Fe-S protein 4, 18kDa (NADH-coenzyme Q reductase) |
| NDUFS5 | NADH dehydrogenase (ubiquinone) Fe-S protein 5, 15kDa (NADH-coenzyme Q reductase) |
| NDUFS6 | NADH dehydrogenase (ubiquinone) Fe-S protein 6, 13kDa (NADH-coenzyme Q reductase) |
| NDUFS7 | NADH dehydrogenase (ubiquinone) Fe-S protein 7, 20kDa (NADH-coenzyme Q reductase) |
| NDUFS8 | NADH dehydrogenase (ubiquinone) Fe-S protein 8, 23kDa (NADH-coenzyme Q reductase) |
| NDUFV1 | NADH dehydrogenase (ubiquinone) flavoprotein 1, 51kDa |
| NDUFV2 | NADH dehydrogenase (ubiquinone) flavoprotein 2, 24kDa |
| NDUFV3 | NADH dehydrogenase (ubiquinone) flavoprotein 3, 10kDa |
| NEU4 | sialidase 4 |
| NFS1 | NFS1 cysteine desulfurase |
| NFU1 | NFU1 iron-sulfur cluster scaffold |
| NGRN | neugrin, neurite outgrowth associated |
| NIF3L1 | NIF3 NGG1 interacting factor 3-like 1 |
| NIPSNAP1 | nipsnap homolog 1 (C. elegans) |
| NIPSNAP2 | nipsnap homolog 2 |
| NIPSNAP3A | nipsnap homolog 3A (C. elegans) |
| NIPSNAP3B | nipsnap homolog 3B |
| NIT1 | nitrilase 1 |
| NIT2 | nitrilase family member 2 |
| NLN | neurolysin (metallopeptidase M3 family) |
| NLRX1 | NLR family member X1 |
| NME3 | NME/NM23 nucleoside diphosphate kinase 3 |
| NME4 | NME/NM23 nucleoside diphosphate kinase 4 |
| NME6 | NME/NM23 nucleoside diphosphate kinase 6 |
| NMNAT3 | nicotinamide nucleotide adenylyltransferase 3 |
| NNT | nicotinamide nucleotide transhydrogenase |
| NOA1 | nitric oxide associated 1 |
| NOCT | nocturnin |
| NOL3 | nucleolar protein 3 (apoptosis repressor with CARD domain) |
| NOX4 | NADPH oxidase 4 |
| NQO1 | NAD(P)H dehydrogenase, quinone 1 |
| NR3C1 | nuclear receptor subfamily 3, group C, member 1 (glucocorticoid receptor) |
| NRD1 | nardilysin (N-arginine dibasic convertase) |
| NRDC | nardilysin convertase |
| NRP1 | neuropilin 1 |
| NSUN2 | NOP2/Sun RNA methyltransferase family, member 2 |
| NSUN3 | NOP2/Sun domain family, member 3 |
| NSUN4 | NOP2/Sun domain family, member 4 |
| NT5DC2 | 5-nucleotidase domain containing 2 |
| NT5DC3 | 5-nucleotidase domain containing 3 |
| NT5M | 5,3-nucleotidase, mitochondrial |
| NTHL1 | nth like DNA glycosylase 1 |
| NUBPL | nucleotide binding protein-like |
| NUCB2 | nucleobindin 2 |
| NUDT1 | nudix (nucleoside diphosphate linked moiety X)-type motif 1 |
| NUDT13 | nudix (nucleoside diphosphate linked moiety X)-type motif 13 |
| NUDT19 | nudix (nucleoside diphosphate linked moiety X)-type motif 19 |
| NUDT2 | nudix hydrolase 2 |
| NUDT5 | nudix hydrolase 5 |
| NUDT6 | nudix (nucleoside diphosphate linked moiety X)-type motif 6 |
| NUDT8 | nudix (nucleoside diphosphate linked moiety X)-type motif 8 |
| NUDT9 | nudix (nucleoside diphosphate linked moiety X)-type motif 9 |
| OAS1 | 2-5-oligoadenylate synthetase 1, 40/46kDa |
| OAT | ornithine aminotransferase |
| OCIAD1 | OCIA domain containing 1 |
| OCIAD2 | OCIA domain containing 2 |
| OGDH | oxoglutarate (alpha-ketoglutarate) dehydrogenase (lipoamide) |
| OGDHL | oxoglutarate dehydrogenase-like |
| OGG1 | 8-oxoguanine DNA glycosylase |
| OGT | O-linked N-acetylglucosamine (GlcNAc) transferase |
| OMA1 | OMA1 zinc metallopeptidase |
| OPA1 | optic atrophy 1 (autosomal dominant) |
| OPA3 | optic atrophy 3 (autosomal recessive, with chorea and spastic paraplegia) |
| OSBPL1A | oxysterol binding protein like 1A |
| OSGEPL1 | O-sialoglycoprotein endopeptidase-like 1 |
| OTC | ornithine carbamoyltransferase |
| OTX2 | orthodenticle homeobox 2 |
| OXA1L | oxidase (cytochrome c) assembly 1-like |
| OXCT1 | 3-oxoacid CoA transferase 1 |
| OXCT2 | 3-oxoacid CoA transferase 2 |
| OXLD1 | oxidoreductase like domain containing 1 |
| OXNAD1 | oxidoreductase NAD-binding domain containing 1 |
| OXR1 | oxidation resistance 1 |
| OXSM | 3-oxoacyl-ACP synthase, mitochondrial |
| P2RY1 | purinergic receptor P2Y, G-protein coupled, 1 |
| P2RY12 | purinergic receptor P2Y, G-protein coupled, 12 |
| P4HB | prolyl 4-hydroxylase, beta polypeptide |
| PABPC5 | poly(A) binding protein, cytoplasmic 5 |
| PACS2 | phosphofurin acidic cluster sorting protein 2 |
| PAICS | phosphoribosylaminoimidazole carboxylase, phosphoribosylaminoimidazole succinocarboxamide synthetase |
| PAK7 | p21 protein (Cdc42/Rac)-activated kinase 7 |
| PAM16 | presequence translocase-associated motor 16 homolog (S. cerevisiae) |
| PANK2 | pantothenate kinase 2 |
| PAPSS2 | 3-phosphoadenosine 5-phosphosulfate synthase 2 |
| PARK7 | parkinson protein 7 |
| PARL | presenilin associated, rhomboid-like |
| PARP1 | poly (ADP-ribose) polymerase 1 |
| PARS2 | prolyl-tRNA synthetase 2, mitochondrial (putative) |
| PC | pyruvate carboxylase |
| PCBD2 | pterin-4 alpha-carbinolamine dehydratase 2 |
| PCCA | propionyl CoA carboxylase, alpha polypeptide |
| PCCB | propionyl CoA carboxylase, beta polypeptide |
| PCK2 | phosphoenolpyruvate carboxykinase 2 (mitochondrial) |
| PDE12 | phosphodiesterase 12 |
| PDE2A | phosphodiesterase 2A, cGMP-stimulated |
| PDF | peptide deformylase (mitochondrial) |
| PDHA1 | pyruvate dehydrogenase (lipoamide) alpha 1 |
| PDHA2 | pyruvate dehydrogenase (lipoamide) alpha 2 |
| PDHB | pyruvate dehydrogenase (lipoamide) beta |
| PDHX | pyruvate dehydrogenase complex, component X |
| PDK1 | pyruvate dehydrogenase kinase, isozyme 1 |
| PDK2 | pyruvate dehydrogenase kinase, isozyme 2 |
| PDK3 | pyruvate dehydrogenase kinase, isozyme 3 |
| PDK4 | pyruvate dehydrogenase kinase, isozyme 4 |
| PDP1 | pyruvate dehyrogenase phosphatase catalytic subunit 1 |
| PDP2 | pyruvate dehyrogenase phosphatase catalytic subunit 2 |
| PDPR | pyruvate dehydrogenase phosphatase regulatory subunit |
| PDSS1 | prenyl (decaprenyl) diphosphate synthase, subunit 1 |
| PDSS2 | prenyl (decaprenyl) diphosphate synthase, subunit 2 |
| PET100 | PET100 homolog |
| PET117 | PET117 homolog |
| PEX11B | peroxisomal biogenesis factor 11 beta |
| PEX3 | peroxisomal biogenesis factor 3 |
| PGAM5 | phosphoglycerate mutase family member 5 |
| PGK1 | phosphoglycerate kinase 1 |
| PGS1 | phosphatidylglycerophosphate synthase 1 |
| PHB | prohibitin |
| PHB2 | prohibitin 2 |
| PHYH | phytanoyl-CoA 2-hydroxylase |
| PHYKPL | 5-phosphohydroxy-L-lysine phospho-lyase |
| PICK1 | protein interacting with PRKCA 1 |
| PIF1 | PIF1 5-to-3 DNA helicase |
| PIGBOS1 | PIGB opposite strand 1 |
| PIN4 | peptidylprolyl cis/trans isomerase, NIMA-interacting 4 |
| PINK1 | PTEN induced putative kinase 1 |
| PISD | phosphatidylserine decarboxylase |
| PITRM1 | pitrilysin metallopeptidase 1 |
| PLD6 | phospholipase D family, member 6 |
| PLGRKT | plasminogen receptor, C-terminal lysine transmembrane protein |
| PLIN5 | perilipin 5 |
| PLPBP | pyridoxal phosphate binding protein |
| PLSCR3 | phospholipid scramblase 3 |
| PMAIP1 | phorbol-12-myristate-13-acetate-induced protein 1 |
| PMPCA | peptidase (mitochondrial processing) alpha |
| PMPCB | peptidase (mitochondrial processing) beta |
| PNKD | paroxysmal nonkinesigenic dyskinesia |
| PNKP | polynucleotide kinase 3-phosphatase |
| PNPLA4 | patatin-like phospholipase domain containing 4 |
| PNPLA8 | patatin-like phospholipase domain containing 8 |
| PNPO | pyridoxamine 5-phosphate oxidase |
| PNPT1 | polyribonucleotide nucleotidyltransferase 1 |
| POLB | DNA polymerase beta |
| POLDIP2 | polymerase (DNA-directed), delta interacting protein 2 |
| POLG | polymerase (DNA directed), gamma |
| POLG2 | polymerase (DNA directed), gamma 2, accessory subunit |
| POLQ | DNA polymerase theta |
| POLRMT | polymerase (RNA) mitochondrial (DNA directed) |
| PON2 | paraoxonase 2 |
| PPA2 | pyrophosphatase (inorganic) 2 |
| PPAN | peter pan homolog (Drosophila) |
| PPIF | peptidylprolyl isomerase F |
| PPL | periplakin |
| PPM1K | protein phosphatase, Mg2+/Mn2+ dependent, 1K |
| PPOX | protoporphyrinogen oxidase |
| PPP1R15A | protein phosphatase 1, regulatory subunit 15A |
| PPTC7 | PTC7 protein phosphatase homolog |
| PRDX2 | peroxiredoxin 2 |
| PRDX3 | peroxiredoxin 3 |
| PRDX4 | peroxiredoxin 4 |
| PRDX5 | peroxiredoxin 5 |
| PRDX6 | peroxiredoxin 6 |
| PRELID1 | PRELI domain containing 1 |
| PRELID2 | PRELI domain containing 2 |
| PRELID3A | PRELI domain containing 3A |
| PRELID3B | PRELI domain containing 3B |
| PREPL | prolyl endopeptidase-like |
| PRICKLE3 | prickle homolog 3 |
| PRIMPOL | primase and polymerase (DNA-directed) |
| PRKACA | protein kinase, cAMP-dependent, catalytic, alpha |
| PRKCE | protein kinase C, epsilon |
| PRKN | parkin RBR E3 ubiquitin protein ligase |
| PRODH | proline dehydrogenase (oxidase) 1 |
| PRODH2 | proline dehydrogenase (oxidase) 2 |
| PRORP | protein only RNase P catalytic subunit |
| PROSC | proline synthetase co-transcribed homolog (bacterial) |
| PRSS35 | serine protease 35 |
| PRXL2A | peroxiredoxin like 2A |
| PSEN1 | presenilin 1 |
| PTCD1 | pentatricopeptide repeat domain 1 |
| PTCD2 | pentatricopeptide repeat domain 2 |
| PTCD3 | pentatricopeptide repeat domain 3 |
| PTGES2 | prostaglandin E synthase 2 |
| PTPMT1 | protein tyrosine phosphatase, mitochondrial 1 |
| PTPN11 | protein tyrosine phosphatase, non-receptor type 11 |
| PTRH1 | peptidyl-tRNA hydrolase 1 homolog |
| PTRH2 | peptidyl-tRNA hydrolase 2 |
| PUS1 | pseudouridylate synthase 1 |
| PUS10 | pseudouridylate synthase 10 |
| PUSL1 | pseudouridylate synthase-like 1 |
| PXMP2 | peroxisomal membrane protein 2 |
| PXMP4 | peroxisomal membrane protein 4 |
| PYCARD | PYD and CARD domain containing |
| PYCR1 | pyrroline-5-carboxylate reductase 1 |
| PYCR2 | pyrroline-5-carboxylate reductase family, member 2 |
| PYROXD2 | pyridine nucleotide-disulphide oxidoreductase domain 2 |
| PYURF | PIGY upstream reading frame |
| QDPR | quinoid dihydropteridine reductase |
| QRSL1 | glutaminyl-tRNA synthase (glutamine-hydrolyzing)-like 1 |
| QTRT1 | queuine tRNA-ribosyltransferase 1 |
| QTRTD1 | queuine tRNA-ribosyltransferase domain containing 1 |
| RAB10 | RAB10, member RAS oncogene family |
| RAB11B | RAB11B, member RAS oncogene family |
| RAB11FIP5 | RAB11 family interacting protein 5 (class I) |
| RAB24 | RAB24, member RAS oncogene family |
| RAB32 | RAB32, member RAS oncogene family |
| RAB35 | RAB35, member RAS oncogene family |
| RAB3A | RAB3A, member RAS oncogene family |
| RAB3D | RAB3D, member RAS oncogene family |
| RAB40AL | RAB40A, member RAS oncogene family-like |
| RAB5C | RAB5C, member RAS oncogene family |
| RAB5IF | RAB5 interacting factor |
| RAB7A | RAB7A, member RAS oncogene family |
| RAD51 | RAD51 recombinase |
| RAD51C | RAD51 paralog C |
| RALA | v-ral simian leukemia viral oncogene homolog A (ras related) |
| RANBP2 | RAN binding protein 2 |
| RANBP6 | RAN binding protein 6 |
| RARS2 | arginyl-tRNA synthetase 2, mitochondrial |
| RBFA | ribosome binding factor A (putative) |
| RCC1L | RCC1 like |
| RCN2 | reticulocalbin 2, EF-hand calcium binding domain |
| RDH13 | retinol dehydrogenase 13 (all-trans/9-cis) |
| RDH14 | retinol dehydrogenase 14 |
| RECQL4 | RecQ protein-like 4 |
| REXO2 | RNA exonuclease 2 |
| RFK | riboflavin kinase |
| RHOT1 | ras homolog family member T1 |
| RHOT2 | ras homolog family member T2 |
| RICTOR | RPTOR independent companion of MTOR, complex 2 |
| RIDA | reactive intermediate imine deaminase A homolog |
| RIPK3 | receptor-interacting serine-threonine kinase 3 |
| RMDN1 | regulator of microtubule dynamics 1 |
| RMDN3 | regulator of microtubule dynamics 3 |
| RMND1 | required for meiotic nuclear division 1 homolog |
| RNASEH1 | ribonuclease H1 |
| RNASEL | ribonuclease L (2,5-oligoisoadenylate synthetase-dependent) |
| RNASET2 | ribonuclease T2 |
| RNF185 | ring finger protein 185 |
| RNMTL1 | RNA methyltransferase like 1 |
| ROMO1 | reactive oxygen species modulator 1 |
| RP11_469A15.2 | RP11_469A15.2 |
| RPIA | ribose 5-phosphate isomerase A |
| RPL10 | ribosomal protein L10 |
| RPL11 | ribosomal protein L11 |
| RPP14 | Hydroxyacyl-thioester dehydratase type 2, mitochondrial |
| RPS3 | ribosomal protein S3 |
| RPS6KB1 | ribosomal protein S6 kinase, 70kDa, polypeptide 1 |
| RPUSD3 | RNA pseudouridylate synthase domain containing 3 |
| RPUSD4 | RNA pseudouridylate synthase domain containing 4 |
| RSAD1 | radical S-adenosyl methionine domain containing 1 |
| RTL10 | retrotransposon Gag like 10 |
| RTN4IP1 | reticulon 4 interacting protein 1 |
| RUVBL2 | RuvB-like AAA ATPase 2 |
| SACS | sacsin molecular chaperone |
| SAMM50 | SAMM50 sorting and assembly machinery component |
| SAR1A | secretion associated, Ras related GTPase 1A |
| SAR1B | secretion associated, Ras related GTPase 1B |
| SARDH | sarcosine dehydrogenase |
| SARM1 | sterile alpha and TIR motif containing 1 |
| SARS2 | seryl-tRNA synthetase 2, mitochondrial |
| SAT2 | spermidine/spermine N1-acetyltransferase family member 2 |
| SCCPDH | saccharopine dehydrogenase (putative) |
| SCO1 | SCO1 cytochrome c oxidase assembly protein |
| SCO2 | SCO2 cytochrome c oxidase assembly protein |
| SCP2 | sterol carrier protein 2 |
| SDHA | succinate dehydrogenase complex, subunit A, flavoprotein (Fp) |
| SDHAF1 | succinate dehydrogenase complex assembly factor 1 |
| SDHAF2 | succinate dehydrogenase complex assembly factor 2 |
| SDHAF3 | succinate dehydrogenase complex assembly factor 3 |
| SDHAF4 | succinate dehydrogenase complex assembly factor 4 |
| SDHB | succinate dehydrogenase complex, subunit B, iron sulfur (Ip) |
| SDHC | succinate dehydrogenase complex, subunit C, integral membrane protein, 15kDa |
| SDHD | succinate dehydrogenase complex, subunit D, integral membrane protein |
| SDR39U1 | short chain dehydrogenase/reductase family 39U member 1 |
| SDSL | serine dehydratase like |
| SECISBP2 | SECIS binding protein 2 |
| SELENOO | selenoprotein O |
| SELO | selenoprotein O |
| SEPT4 | septin 4 |
| SEPTIN4 | septin 4 |
| SERAC1 | serine active site containing 1 |
| SERHL2 | serine hydrolase like 2 |
| SETD9 | SET domain containing 9 |
| SFXN1 | sideroflexin 1 |
| SFXN2 | sideroflexin 2 |
| SFXN3 | sideroflexin 3 |
| SFXN4 | sideroflexin 4 |
| SFXN5 | sideroflexin 5 |
| SGK1 | serum/glucocorticoid regulated kinase 1 |
| SH3BP5 | SH3-domain binding protein 5 (BTK-associated) |
| SH3GLB1 | SH3-domain GRB2-like endophilin B1 |
| SHC1 | SHC (Src homology 2 domain containing) transforming protein 1 |
| SHMT2 | serine hydroxymethyltransferase 2 (mitochondrial) |
| SIAH1 | siah E3 ubiquitin protein ligase 1 |
| SIAH3 | siah E3 ubiquitin protein ligase family member 3 |
| SIRT3 | sirtuin 3 |
| SIRT4 | sirtuin 4 |
| SIRT5 | sirtuin 5 |
| SLC11A2 | solute carrier family 11 (proton-coupled divalent metal ion transporter), member 2 |
| SLC22A4 | solute carrier family 22 (organic cation/zwitterion transporter), member 4 |
| SLC24A5 | solute carrier family 24 (sodium/potassium/calcium exchanger), member 5 |
| SLC25A1 | solute carrier family 25 (mitochondrial carrier; citrate transporter), member 1 |
| SLC25A10 | solute carrier family 25 (mitochondrial carrier; dicarboxylate transporter), member 10 |
| SLC25A11 | solute carrier family 25 (mitochondrial carrier; oxoglutarate carrier), member 11 |
| SLC25A12 | solute carrier family 25 (aspartate/glutamate carrier), member 12 |
| SLC25A13 | solute carrier family 25 (aspartate/glutamate carrier), member 13 |
| SLC25A14 | solute carrier family 25 (mitochondrial carrier, brain), member 14 |
| SLC25A15 | solute carrier family 25 (mitochondrial carrier; ornithine transporter) member 15 |
| SLC25A16 | solute carrier family 25 (mitochondrial carrier), member 16 |
| SLC25A18 | solute carrier family 25 (glutamate carrier), member 18 |
| SLC25A19 | solute carrier family 25 (mitochondrial thiamine pyrophosphate carrier), member 19 |
| SLC25A2 | solute carrier family 25 (mitochondrial carrier; ornithine transporter) member 2 |
| SLC25A20 | solute carrier family 25 (carnitine/acylcarnitine translocase), member 20 |
| SLC25A21 | solute carrier family 25 (mitochondrial oxoadipate carrier), member 21 |
| SLC25A22 | solute carrier family 25 (mitochondrial carrier: glutamate), member 22 |
| SLC25A23 | solute carrier family 25 (mitochondrial carrier; phosphate carrier), member 23 |
| SLC25A24 | solute carrier family 25 (mitochondrial carrier; phosphate carrier), member 24 |
| SLC25A25 | solute carrier family 25 (mitochondrial carrier; phosphate carrier), member 25 |
| SLC25A26 | solute carrier family 25 (S-adenosylmethionine carrier), member 26 |
| SLC25A27 | solute carrier family 25, member 27 |
| SLC25A28 | solute carrier family 25 (mitochondrial iron transporter), member 28 |
| SLC25A29 | solute carrier family 25 (mitochondrial carnitine/acylcarnitine carrier), member 29 |
| SLC25A3 | solute carrier family 25 (mitochondrial carrier; phosphate carrier), member 3 |
| SLC25A30 | solute carrier family 25, member 30 |
| SLC25A31 | solute carrier family 25 (mitochondrial carrier; adenine nucleotide translocator), member 31 |
| SLC25A32 | solute carrier family 25 (mitochondrial folate carrier), member 32 |
| SLC25A33 | solute carrier family 25 (pyrimidine nucleotide carrier), member 33 |
| SLC25A34 | solute carrier family 25, member 34 |
| SLC25A35 | solute carrier family 25, member 35 |
| SLC25A36 | solute carrier family 25 (pyrimidine nucleotide carrier), member 36 |
| SLC25A37 | solute carrier family 25 (mitochondrial iron transporter), member 37 |
| SLC25A38 | solute carrier family 25, member 38 |
| SLC25A39 | solute carrier family 25, member 39 |
| SLC25A4 | solute carrier family 25 (mitochondrial carrier; adenine nucleotide translocator), member 4 |
| SLC25A40 | solute carrier family 25, member 40 |
| SLC25A41 | solute carrier family 25, member 41 |
| SLC25A42 | solute carrier family 25, member 42 |
| SLC25A43 | solute carrier family 25, member 43 |
| SLC25A44 | solute carrier family 25, member 44 |
| SLC25A45 | solute carrier family 25, member 45 |
| SLC25A46 | solute carrier family 25, member 46 |
| SLC25A47 | solute carrier family 25, member 47 |
| SLC25A48 | solute carrier family 25, member 48 |
| SLC25A5 | solute carrier family 25 (mitochondrial carrier; adenine nucleotide translocator), member 5 |
| SLC25A51 | solute carrier family 25, member 51 |
| SLC25A52 | solute carrier family 25, member 52 |
| SLC25A53 | solute carrier family 25, member 53 |
| SLC25A6 | solute carrier family 25 (mitochondrial carrier; adenine nucleotide translocator), member 6 |
| SLC30A9 | solute carrier family 30 (zinc transporter), member 9 |
| SLC35F6 | solute carrier family 35, member F6 |
| SLC44A1 | solute carrier family 44 (choline transporter), member 1 |
| SLC8A3 | solute carrier family 8 (sodium/calcium exchanger), member 3 |
| SLC8B1 | solute carrier family 8 (sodium/lithium/calcium exchanger), member B1 |
| SLC9A6 | solute carrier family 9, subfamily A (NHE6, cation proton antiporter 6), member 6 |
| SLC9B2 | solute carrier family 9, subfamily B (NHA2, cation proton antiporter 2), member 2 |
| SLIRP | SRA stem-loop interacting RNA binding protein |
| SLIT3 | slit guidance ligand 3 |
| SLMO1 | slowmo homolog 1 (Drosophila) |
| SLMO2 | slowmo homolog 2 (Drosophila) |
| SMCP | sperm mitochondria-associated cysteine-rich protein |
| SMDT1 | single-pass membrane protein with aspartate-rich tail 1 |
| SMIM20 | small integral membrane protein 20 |
| SMIM8 | small integral membrane protein 8 |
| SMURF1 | SMAD specific E3 ubiquitin protein ligase 1 |
| SNAP29 | synaptosomal-associated protein, 29kDa |
| SNCA | synuclein, alpha (non A4 component of amyloid precursor) |
| SND1 | staphylococcal nuclease and tudor domain containing 1 |
| SNN | stannin |
| SNPH | syntaphilin |
| SOD1 | superoxide dismutase 1 |
| SOD2 | superoxide dismutase 2, mitochondrial |
| SPATA18 | spermatogenesis associated 18 |
| SPATA19 | spermatogenesis associated 19 |
| SPATA20 | spermatogenesis associated 20 |
| SPATA5 | spermatogenesis associated 5 |
| SPG20 | spastic paraplegia 20 (Troyer syndrome) |
| SPG7 | spastic paraplegia 7 (pure and complicated autosomal recessive) |
| SPHK2 | sphingosine kinase 2 |
| SPHKAP | SPHK1 interactor, AKAP domain containing |
| SPIRE1 | spire type actin nucleation factor 1 |
| SPNS1 | spinster homolog 1 (Drosophila) |
| SPR | sepiapterin reductase |
| SPRYD4 | SPRY domain containing 4 |
| SPTLC2 | serine palmitoyltransferase long chain base subunit 2 |
| SQOR | sulfide quinone oxidoreductase |
| SQRDL | sulfide quinone reductase-like (yeast) |
| SRC | SRC proto-oncogene, non-receptor tyrosine kinase |
| SSBP1 | single-stranded DNA binding protein 1, mitochondrial |
| STAR | steroidogenic acute regulatory protein |
| STARD13 | StAR-related lipid transfer (START) domain containing 13 |
| STARD7 | StAR-related lipid transfer (START) domain containing 7 |
| STAT3 | signal transducer and activator of transcription 3 (acute-phase response factor) |
| STOM | stomatin |
| STOML2 | stomatin (EPB72)-like 2 |
| STX17 | syntaxin 17 |
| STYXL1 | serine/threonine/tyrosine interacting like 1 |
| SUCLA2 | succinate-CoA ligase, ADP-forming, beta subunit |
| SUCLG1 | succinate-CoA ligase, alpha subunit |
| SUCLG2 | succinate-CoA ligase, GDP-forming, beta subunit |
| SUGCT | succinyl-CoA:glutarate-CoA transferase |
| SUOX | sulfite oxidase |
| SUPV3L1 | suppressor of var1, 3-like 1 (S. cerevisiae) |
| SURF1 | surfeit 1 |
| SYBU | syntabulin (syntaxin-interacting) |
| SYNJ2BP | synaptojanin 2 binding protein |
| TACO1 | translational activator of mitochondrially encoded cytochrome c oxidase I |
| TAMM41 | TAM41, mitochondrial translocator assembly and maintenance protein, homolog (S. cerevisiae) |
| TANGO2 | transport and golgi organization 2 homolog |
| TARS2 | threonyl-tRNA synthetase 2, mitochondrial (putative) |
| TAZ | tafazzin |
| TBC1D15 | TBC1 domain family, member 15 |
| TBC1D9 | TBC1 domain family, member 9 (with GRAM domain) |
| TBRG4 | transforming growth factor beta regulator 4 |
| TCAIM | T cell activation inhibitor, mitochondrial |
| TCHP | trichoplein, keratin filament binding |
| TDRD7 | tudor domain containing 7 |
| TDRKH | tudor and KH domain containing |
| TEFM | transcription elongation factor, mitochondrial |
| TERT | telomerase reverse transcriptase |
| TFAM | transcription factor A, mitochondrial |
| TFB1M | transcription factor B1, mitochondrial |
| TFB2M | transcription factor B2, mitochondrial |
| THEM4 | thioesterase superfamily member 4 |
| THEM5 | thioesterase superfamily member 5 |
| THG1L | tRNA-histidine guanylyltransferase 1-like (S. cerevisiae) |
| THNSL1 | threonine synthase-like 1 (S. cerevisiae) |
| TIGAR | TP53 induced glycolysis regulatory phosphatase |
| TIMM10 | translocase of inner mitochondrial membrane 10 homolog (yeast) |
| TIMM10B | translocase of inner mitochondrial membrane 10 homolog B (yeast) |
| TIMM13 | translocase of inner mitochondrial membrane 13 homolog (yeast) |
| TIMM17A | translocase of inner mitochondrial membrane 17 homolog A (yeast) |
| TIMM17B | translocase of inner mitochondrial membrane 17 homolog B (yeast) |
| TIMM21 | translocase of inner mitochondrial membrane 21 homolog (yeast) |
| TIMM22 | translocase of inner mitochondrial membrane 22 homolog (yeast) |
| TIMM23 | translocase of inner mitochondrial membrane 23 homolog (yeast) |
| TIMM23B | translocase of inner mitochondrial membrane 23 homolog B (yeast) |
| TIMM29 | translocase of inner mitochondrial membrane 29 |
| TIMM44 | translocase of inner mitochondrial membrane 44 homolog (yeast) |
| TIMM50 | translocase of inner mitochondrial membrane 50 homolog (S. cerevisiae) |
| TIMM8A | translocase of inner mitochondrial membrane 8 homolog A (yeast) |
| TIMM8B | translocase of inner mitochondrial membrane 8 homolog B (yeast) |
| TIMM9 | translocase of inner mitochondrial membrane 9 homolog (yeast) |
| TIMMDC1 | translocase of inner mitochondrial membrane domain containing 1 |
| TK2 | thymidine kinase 2, mitochondrial |
| TMEM11 | transmembrane protein 11 |
| TMEM126A | transmembrane protein 126A |
| TMEM126B | transmembrane protein 126B |
| TMEM143 | transmembrane protein 143 |
| TMEM14C | transmembrane protein 14C |
| TMEM177 | transmembrane protein 177 |
| TMEM186 | transmembrane protein 186 |
| TMEM205 | transmembrane protein 205 |
| TMEM223 | transmembrane protein 223 |
| TMEM243 | transmembrane protein 243, mitochondrial |
| TMEM261 | transmembrane protein 261 |
| TMEM263 | transmembrane protein 263 |
| TMEM65 | transmembrane protein 65 |
| TMEM70 | transmembrane protein 70 |
| TMIGD1 | transmembrane and immunoglobulin domain containing 1 |
| TMLHE | trimethyllysine hydroxylase, epsilon |
| TMX1 | thioredoxin-related transmembrane protein 1 |
| TMX2 | thioredoxin-related transmembrane protein 2 |
| TOMM20 | translocase of outer mitochondrial membrane 20 homolog (yeast) |
| TOMM20L | translocase of outer mitochondrial membrane 20 homolog (yeast)-like |
| TOMM22 | translocase of outer mitochondrial membrane 22 homolog (yeast) |
| TOMM34 | translocase of outer mitochondrial membrane 34 |
| TOMM40 | translocase of outer mitochondrial membrane 40 homolog (yeast) |
| TOMM40L | translocase of outer mitochondrial membrane 40 homolog (yeast)-like |
| TOMM5 | translocase of outer mitochondrial membrane 5 homolog (yeast) |
| TOMM6 | translocase of outer mitochondrial membrane 6 homolog (yeast) |
| TOMM7 | translocase of outer mitochondrial membrane 7 homolog (yeast) |
| TOMM70 | translocase of outer mitochondrial membrane 70 |
| TOMM70A | translocase of outer mitochondrial membrane 70 homolog A (S. cerevisiae) |
| TOP1MT | topoisomerase (DNA) I, mitochondrial |
| TOP3A | topoisomerase (DNA) III alpha |
| TP53AIP1 | tumor protein p53 regulated apoptosis inducing protein 1 |
| TRABD | TraB domain containing |
| TRAF6 | TNF receptor-associated factor 6, E3 ubiquitin protein ligase |
| TRAK1 | trafficking protein, kinesin binding 1 |
| TRAP1 | TNF receptor-associated protein 1 |
| TRIAP1 | TP53 regulated inhibitor of apoptosis 1 |
| TRIT1 | tRNA isopentenyltransferase 1 |
| TRMT1 | tRNA methyltransferase 1 homolog (S. cerevisiae) |
| TRMT10C | tRNA methyltransferase 10 homolog C (S. cerevisiae) |
| TRMT11 | tRNA methyltransferase 11 homolog (S. cerevisiae) |
| TRMT2B | tRNA methyltransferase 2 homolog B (S. cerevisiae) |
| TRMT5 | tRNA methyltransferase 5 |
| TRMT61B | tRNA methyltransferase 61B |
| TRMU | tRNA 5-methylaminomethyl-2-thiouridylate methyltransferase |
| TRNT1 | tRNA nucleotidyl transferase, CCA-adding, 1 |
| TRUB2 | TruB pseudouridine (psi) synthase family member 2 |
| TSFM | Ts translation elongation factor, mitochondrial |
| TSPO | translocator protein (18kDa) |
| TST | thiosulfate sulfurtransferase (rhodanese) |
| TSTD1 | thiosulfate sulfurtransferase like domain containing 1 |
| TSTD3 | thiosulfate sulfurtransferase like domain containing 3 |
| TTC19 | tetratricopeptide repeat domain 19 |
| TUBB3 | tubulin, beta 3 class III |
| TUFM | Tu translation elongation factor, mitochondrial |
| TUSC2 | tumor suppressor candidate 2 |
| TWNK | twinkle mtDNA helicase |
| TXN2 | thioredoxin 2 |
| TXNDC12 | thioredoxin domain containing 12 (endoplasmic reticulum) |
| TXNRD1 | thioredoxin reductase 1 |
| TXNRD2 | thioredoxin reductase 2 |
| TYMS | thymidylate synthetase |
| UBA1 | ubiquitin-like modifier activating enzyme 1 |
| UBE2N | ubiquitin-conjugating enzyme E2N |
| UBE3B | ubiquitin protein ligase E3B |
| UBIAD1 | UbiA prenyltransferase domain containing 1 |
| UCP1 | uncoupling protein 1 (mitochondrial, proton carrier) |
| UCP2 | uncoupling protein 2 (mitochondrial, proton carrier) |
| UCP3 | uncoupling protein 3 (mitochondrial, proton carrier) |
| UNG | uracil DNA glycosylase |
| UQCC1 | ubiquinol-cytochrome c reductase complex assembly factor 1 |
| UQCC2 | ubiquinol-cytochrome c reductase complex assembly factor 2 |
| UQCC3 | ubiquinol-cytochrome c reductase complex assembly factor 3 |
| UQCR10 | ubiquinol-cytochrome c reductase, complex III subunit X |
| UQCR11 | ubiquinol-cytochrome c reductase, complex III subunit XI |
| UQCRB | ubiquinol-cytochrome c reductase binding protein |
| UQCRC1 | ubiquinol-cytochrome c reductase core protein I |
| UQCRC2 | ubiquinol-cytochrome c reductase core protein II |
| UQCRFS1 | ubiquinol-cytochrome c reductase, Rieske iron-sulfur polypeptide 1 |
| UQCRH | ubiquinol-cytochrome c reductase hinge protein |
| UQCRQ | ubiquinol-cytochrome c reductase, complex III subunit VII, 9.5kDa |
| USMG5 | up-regulated during skeletal muscle growth 5 homolog (mouse) |
| USP30 | ubiquitin specific peptidase 30 |
| VAMP1 | vesicle-associated membrane protein 1 (synaptobrevin 1) |
| VARS2 | valyl-tRNA synthetase 2, mitochondrial |
| VCP | valosin containing protein |
| VDAC1 | voltage-dependent anion channel 1 |
| VDAC2 | voltage-dependent anion channel 2 |
| VDAC3 | voltage-dependent anion channel 3 |
| VIM | vimentin |
| VPS13A | vacuolar protein sorting 13 homolog A (S. cerevisiae) |
| VPS13D | vacuolar protein sorting 13 homolog D (S. cerevisiae) |
| VRK2 | vaccinia related kinase 2 |
| VWA8 | von Willebrand factor A domain containing 8 |
| WARS2 | tryptophanyl tRNA synthetase 2, mitochondrial |
| WASF1 | WAS protein family, member 1 |
| WBSCR16 | Williams-Beuren syndrome chromosome region 16 |
| WDR81 | WD repeat domain 81 |
| WDR82 | WD repeat domain 82 |
| XAF1 | XIAP associated factor 1 |
| XPNPEP3 | X-prolyl aminopeptidase 3, mitochondrial |
| XRCC3 | X-ray repair complementing defective repair in Chinese hamster cells 3 |
| YARS2 | tyrosyl-tRNA synthetase 2, mitochondrial |
| YBEY | ybeY metallopeptidase (putative) |
| YME1L1 | YME1-like 1 ATPase |
| YRDC | yrdC N(6)-threonylcarbamoyltransferase domain containing |
| ZADH2 | zinc binding alcohol dehydrogenase domain containing 2 |
| ZDHHC8 | zinc finger, DHHC-type containing 8 |
| ZNFX1 | zinc finger, NFX1-type containing 1 |
